# Quantum-Assisted Variational Monte Carlo

**DOI:** 10.1021/prechem.5c00025

**Published:** 2025-06-07

**Authors:** Longfei Chang, Zhendong Li, Wei-Hai Fang

**Affiliations:** Key Laboratory of Theoretical and Computational Photochemistry, Ministry of Education, College of Chemistry, 47836 Beijing Normal University, Beijing 100875, China

**Keywords:** quantum algorithms, strongly correlated systems, variational Monte Carlo, neural-network quantum states, quantum-enhanced Markov chain Monte Carlo

## Abstract

Solving the ground state of quantum many-body systems remains a fundamental challenge in physics and chemistry. Recent advancements in quantum hardware have opened new avenues for addressing this challenge. Inspired by the quantum-enhanced Markov chain Monte Carlo (QeMCMC) algorithm, which was originally designed for sampling the Boltzmann distribution of classical spin models using quantum computers, we introduce a quantum-assisted variational Monte Carlo (QA-VMC) algorithm for solving the ground state of quantum many-body systems by adapting QeMCMC to sample the distribution of a (neural-network) wave function in VMC. The central question is whether such a quantum-assisted proposal can potentially offer a computational advantage over classical methods. Through numerical investigations for the Fermi–Hubbard model and molecular systems, we demonstrate that the quantum-assisted proposal exhibits larger absolute spectral gaps and reduced autocorrelation times compared to conventional classical proposals, leading to more efficient sampling and faster convergence to the ground state in VMC as well as a more accurate and precise estimation of physical observables. This advantage is especially pronounced for specific parameter ranges, where the ground-state configurations are more concentrated in some configurations separated by large Hamming distances. Our results underscore the potential of quantum-assisted algorithms to enhance classical variational methods for solving the ground state of quantum many-body systems.

## Graphical abstract

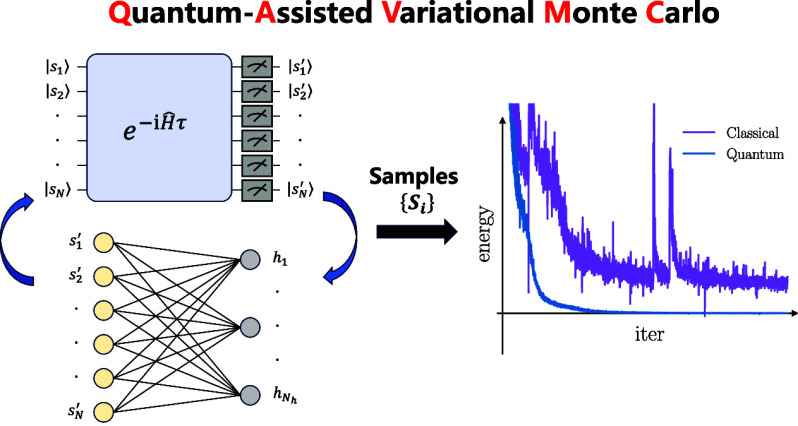

## Introduction

Accurately and efficiently solving the Schrödinger equation continues to pose a great challenge in quantum chemistry and condensed matter physics,^1^ primarily due to the exponential growth of the Hilbert space with increasing system size. To address this fundamental issue, a variety of classical computational methods have been developed, including density functional theory^2–4^ (DFT), coupled cluster theory^5–8^ (CC), density matrix renormalization group^9,10^ (DMRG), and various quantum Monte Carlo^11–15^ (QMC) algorithms. Among these, variational Monte Carlo^16^ (VMC) has attracted significant attention in the era of artificial intelligence,^14,15^ particularly as neural networks (NNs) have emerged as a promising class of variational wave functions. Carleo and Troyer^14^ first employed the restricted Boltzmann machines (RBM), a class of powerful energy-based models widely employed in machine learning for approximating discrete probability distributions,^17^ as variational ansatz for spin systems and achieved high accuracy comparable to that of tensor network methods. This work has inspired subsequent research employing other machine learning models, such as convolutional neural networks (CNNs),^18,19^ autoregressive models,^20–22^ and Transformers,^23–25^ to tackle quantum many-body problems formulated in the framework of second quantization. For related studies addressing the solution of the Schrödinger equation using NNs within the first quantization framework, we refer the reader to ref 26 and the references cited therein. These advancements highlight the growing synergy between VMC and machine learning, offering new avenues for solving complex quantum systems in physics and chemistry.

A key step in VMC is sampling configurations from the probability distribution of the trial wave functions. The Markov chain Monte Carlo (MCMC) algorithm is one of the most widely used methods for this purpose.^27^ However, it may face difficulties, such as prolonged mixing times, in challenging situations. For instance, in classical systems at critical points, the critical slowing down^28^ can significantly increase the mixing time of the Markov chain, making sampling inefficient. Similar problems may also happen in sampling the ground-state distribution of quantum systems, such that a larger number of samples is required to achieve accurate energy estimates, thereby reducing the overall efficiency of the VMC algorithm.^29^ To address these limitations, autoregressive neural networks have emerged as a promising alternative. By parametrizing the electronic wave function using autoregressive architectures,^20–22^ efficient and scalable sampling based on conditional distribution can be achieved without relying on MCMC. However, many previously mentioned NN wave functions without such autoregressive structure, including RBM, CNNs, and vision transformers^24,25^ (ViTs), still rely on MCMC for sampling. Therefore, there persists a critical need for developing innovative strategies to enhance sampling efficiency in VMC.

Thanks to the rapid development of quantum hardware,^30,31^ quantum computation has become a promising tool for tackling challenging computational problems.^32–35^ Many quantum algorithms have been proposed to accelerate sampling from the Gibbs state or the classical Boltzman distribution.^36–53^ In particular, the recently proposed quantum-enhanced Markov chain Monte Carlo (QeMCMC) algorithm^51^ stands out as a hybrid quantum-classical method for sampling from the Boltzmann distribution of classical spin systems, which has been shown to accelerate the convergence of Markov chain for spin-glass models at low temperatures both numerically and experimentally on near-term quantum devices.^51^ This work has spurred several further developments,^52,54–59^ including investigations into the limitations of the algorithm,^54,55^ the use of quantum alternating operator ansatz as an alternative to time evolution to reduce circuit depth,^52^ and the development of quantum-inspired sampling algorithms based on QeMCMC,^56^ and improving sampling efficiency of VMC through surrogate models.^59^

In this work, inspired by the QeMCMC algorithm^51^ for sampling classical Boltzmann distributions, we propose a quantum-assisted VMC (QA-VMC) algorithm to address the sampling challenges for solving quantum many-body problems using VMC. Similar to QeMCMC, our approach leverages the unique capability of quantum computers to perform time evolution and utilizes the resulting quantum states to propose new configurations, while all other components of the algorithm are executed on classical computers to minimize the demand for quantum resources. A central question we aim to explore in this work is whether QA-VMC can offer a potential advantage in sampling the ground state distributions of quantum many-body systems. To investigate this, we benchmark the algorithm against classical sampling methods for various models, including the Fermi–Hubbard model (FHM) and molecular systems, with different system sizes and parameters. The remainder of this article is structured as follows. First, we provide a concise overview of the VMC algorithm and MCMC sampling techniques. Next, we introduce the QA-VMC algorithm and the figures of merit used to evaluate the convergence of the different MCMC algorithms. Subsequently, we present the results of the quantum-assisted algorithm for various systems and compare its performance with classical sampling methods. Finally, we summarize our findings and discuss future directions.

## Theory and Algorithms

### Variational Monte Carlo

The VMC^60–62^ method is a computational algorithm that combines the variational principle with Monte Carlo sampling to approximate the ground state of a Hamiltonian *Ĥ* using a trial wave function. Specifically, for a variational wave function |ψ_**θ**_⟩ characterized by a set of variational parameters **θ**, the energy function can be expressed as

1 $$E_{\theta }=\frac{\left\langle \psi_{\theta }\left|Ĥ\right|\psi_{\theta }\right\rangle }{\langle \psi_{\theta }|\psi_{\theta }\rangle }=\underset{S}{\sum }P_{\theta }\left(S\right)E_{\theta }^{loc}\left(S\right)$$

where the configuration ***S*** ≡ (*s*_1_, ..., *s*_*N*_) consists of spins (or qubits) *s*_*j*_ = ±1. The probability distribution is defined as *P*_**θ**_(***S***) ≡ |⟨ ***S*** |ψ_**θ**_⟩|^2^/⟨ψ_**θ**_|ψ_**θ**_⟩, and the local energy is given by

2 $$E_{\theta }^{loc}\left(S\right)\equiv \frac{\left\langle S\left|Ĥ\right|\psi_{\theta }\right\rangle }{\langle S|\psi_{\theta }\rangle }=\frac{\underset{S^{\prime }}{\sum }\langle S|Ĥ|S^{\prime }\rangle \langle S^{\prime }|\psi_{\theta }\rangle }{\langle S|\psi_{\theta }\rangle }$$

In the VMC framework, the energy function is approximated using the Monte Carlo algorithm by sampling configurations {***S***_*i*_} from *P*_**θ**_(***S***), i.e.

3 $$E_{\theta }\approx \frac{1}{N_{s}}\overset{N_{s}}{\underset{i=1}{\sum }}E_{\theta }^{loc}\left(S_{i}\right)$$

where *N*_*s*_ denotes the number of samples. Similarly, the energy gradients with respect to the parameters can be estimated as^60^

4 $$\frac{\partial E_{\theta }}{\partial \theta }\approx \frac{1}{N_{s}}\overset{N_{s}}{\underset{i=1}{\sum }}2R\left[\left(E_{\theta }^{loc}\left(S_{i}\right)-E_{\theta }\right)\frac{\partial \;\ln\psi_{\theta }^{*}\left(S_{i}\right)}{\partial \theta }\right]$$

For sparse Hamiltonians, the local energy ([Disp-formula eq2]) can be computed with polynomial cost with respect to the system size *N*, provided that the value of the trial wave function ψ_**θ**_(***S***_*i*_) can be evaluated with polynomial cost. Consequently, VMC enables efficient estimation of the energy and optimization of the parameters, even for highly complex wave function ansätze for which the overlap ⟨ψ_**θ**_|ψ_**θ**_⟩ and the expectation value of the Hamiltonian $\left\langle \psi_{\theta }\left|Ĥ\right|\psi_{\theta }\right\rangle$ cannot be efficiently computed exactly.

The accuracy of VMC calculations is strongly dependent on the flexibility of the wave function ansatz. The RBM ansatz^14^ for the wave function |ψ_**θ**_⟩ = *∑*_***S***_ψ_**θ**_(***S***)| ***S*** ⟩ can be expressed as

5 $$\psi_{\theta }\left(S\right)=\underset{h}{\sum }\exp\left(E_{\theta }^{RBM}\left(S\right)\right)$$

6 $$E_{\theta }^{RBM}\left(S\right)=\overset{N}{\underset{i=1}{\sum }}a_{i}s_{i}+\overset{M}{\underset{\mu =1}{\sum }}b_{\mu }h_{\mu }+\overset{N}{\underset{i=1}{\sum }}\overset{M}{\underset{\mu =1}{\sum }}s_{i}W_{\mu i}h_{\mu }$$

where *h*_μ_ ∈ { −1, 1} is a set of binary hidden variables, and the set of real or complex variables **θ** = {*W*_μ*i*_, *a*_*i*_, *b*_μ_} are variational parameters. Here, *W*_μ*i*_ denotes the weights connecting variables *s*_*i*_ and *h*_μ_, and *a*_*i*_ and *b*_μ_ are the biases associated with the physical variables *s*_*i*_ and hidden variables *h*_μ_, respectively. The representational power of RBM increases with the number of hidden variables *M*, and the density of hidden units, defined as α ≡ *M*/*N*, serves as a measure of the model’s complexity. In this work, we utilized the RBMmodPhase ansatz^63^ implemented in the NetKet package^62^ as trial wave functions. This ansatz employs two RBMs with real parameters, denoted by *A*_**θ**_(***S***) and *B*_**ϕ**_(***S***), to separately model the amplitude and phase of the wave function, i.e., $\psi_{\theta ,\phi }\left(S\right)=A_{\theta }\left(S\right)e^{i\;\ln B_{\phi }\left(S\right)}$ . For optimization, we employed the stochastic reconfiguration method^60^ in conjunction with the Adam optimizer.^64^

### Markov Chain Monte Carlo

To sample configurations from probability distribution *P*_**θ**_(***S***), the MCMC algorithm is commonly employed in VMC. MCMC generates samples from a target probability distribution π­(***S***) by constructing a Markov chain that explores a defined state space {***S***_*i*_}. The transition from state ***S***_*i*_ to state ***S***_*j*_ is governed by a transition probability $P\left(S_{i},S_{j}\right)$ . If the Markov chain is *irreducible* and *aperiodic*, it is guaranteed to converge to a unique stationary distribution,^27^ which corresponds to the target distribution π­(***S***). A sufficient condition to ensure this convergence is the detailed balance condition expressed as

7 $$\pi \left(S_{i}\right)P\left(S_{i},S_{j}\right)=\pi \left(S_{j}\right)P\left(S_{j},S_{i}\right),\forall i,j$$

One of the most widely used sampling methods that satisfies the detailed balance condition is the Metropolis–Hastings algorithm.^65^ This algorithm decomposes the transition process into two steps: first, a candidate move is proposed according to a proposal distribution $Q\left(S_{i},S_{j}\right)$ , and second, the move is either accepted or rejected based on an acceptance probability $A\left(S_{i},S_{j}\right)$ , defined as

8 $$A\left(S_{i},S_{j}\right)=\min\left(1,\frac{\pi \left(S_{j}\right)Q\left(S_{j},S_{i}\right)}{\pi \left(S_{i}\right)Q\left(S_{i},Sj\right)}\right)$$

Using this approach, a Markov chain can be constructed for any target probability distribution π­(***S***) on the state space {***S***_*i*_}, with a transition matrix P given by

9 $$P\left(S_{i},S_{j}\right)=\{\begin{array}{ll} Q\left(S_{i},S_{j}\right)A\left(S_{i},S_{j}\right) & \mathrm{if}\;S_{j}\neq S_{i}\\ 1-\underset{S^{\prime }\neq S_{i}}{\sum }Q\left(S_{i},S^{\prime }\right)A\left(S_{i},S^{\prime }\right) & \mathrm{if}\;S_{j}=S_{i} \end{array}$$

The proposal distribution $Q\left(S_{i},S_{j}\right)$ can take nearly any form, provided it is efficiently computable. However, since different $Q\left(S_{i},S_{j}\right)$ will result in different $P\left(S_{i},S_{j}\right)$ , its choice has a significant impact on the convergence rate of the MCMC algorithm. A well-designed proposal distribution can significantly enhance sampling efficiency, enabling a faster exploration of the state space. On the other hand, a poorly chosen proposal distribution may result in slow convergence or inefficient exploration of the state space. For Fermionic systems, such as the FHMs and molecular systems, commonly employed proposals encompass the Uniform proposal (selecting a random configuration), the Exchange proposal (swapping occupations of two same-spin orbitals randomly), and the ExcitationSD proposal, which generates new configurations through restricted random excitations, similar to the Uniform proposal but limited to single and double excitations.

Recently, Layden et al.^51^ introduced the QeMCMC algorithm for sampling from the Boltzmann distribution $\pi \left(S\right)=\frac{1}{Z}e^{-E\left(S\right)/T}$ of the “spin glass” Ising model, where the energy of a configuration ***S*** is given by $E\left(S\right)=-\overset{n}{\underset{j>k=1}{\sum }}J_{jk}s_{j}s_{k}-\overset{n}{\underset{j=1}{\sum }}h_{j}s_{j}$ , with *T* being the temperature and *Z* being the partition function. In this approach, proposals are generated with the help of time evolution on quantum computers. Specifically, the time evolution operator Û(γ,τ)=exp(−iĤ(γ)τ) is constructed from a specially designed Hamiltonian

10 $$Ĥ\left(\gamma \right)=\left(1-\gamma \right)\alpha {Ĥ}_{prob}+\gamma {Ĥ}_{mix}$$

where ${Ĥ}_{prob}$ shares the same parameters with the problem and ${Ĥ}_{mix}$ is a mixing term

11 $${Ĥ}_{prob}=-\overset{n}{\underset{j>k=1}{\sum }}J_{jk}{Ẑ}_{j}{Ẑ}_{k}-\overset{n}{\underset{j=1}{\sum }}h_{j}{Ẑ}_{j}$$

12 $${Ĥ}_{mix}=\overset{n}{\underset{j=1}{\sum }}{\mathrm{X̂}}_{j}$$

Here, $\alpha =∥{Ĥ}_{mix}{∥}_{F}/∥{Ĥ}_{prob}{∥}_{F}$ is a normalizing factor, and γ ∈ [0, 1] controls the relative weights of the two terms. The quantum proposal distribution is then defined as

13 $$Q\left(S_{i},S_{j};\gamma ,\tau \right)=|\langle S_{j}|\exp\left(-iĤ\left(\gamma \right)\tau \right){|S_{i}\rangle |}^{2}$$

In the QeMCMC procedure,^51^ γ and τ are randomly selected within predefined ranges at each MCMC step. Notably, since $Ĥ={Ĥ}^{T}$ in eq 10, it follows that $Û={Û}^{T}$ and $Q=Q^{T}$ . Consequently, the acceptance probability in eq 8 simplifies to

14 $$A\left(S_{i},S_{j}\right)=\min\left(1,\frac{\pi \left(S_{j}\right)}{\pi \left(S_{i}\right)}\right)$$

which avoids the explicit computation of Q . Numerical and experimental results demonstrate that this quantum proposal leads to faster convergence at low temperatures compared to classical local and uniform moves.^51^ This improvement is attributed to the ability of the quantum proposal to generate moves that result in small energy changes |Δ*E*| = |*E*(***S***_*i*_) – *E*(***S***_*j*_)|, while achieving large Hamming distances, thus enhancing exploration efficiency for challenging distributions.

### Quantum-Assisted Variational Monte Carlo

Inspired by the QeMCMC algorithm^51^ for sampling classical Boltzmann distributions, we propose the QA-VMC algorithm, as illustrated in Figure 1, for solving quantum many-body systems. The code is available in ref 66. Given a problem specified by the Hamiltonian *Ĥ*(*x*), which depends on a parameter *x* such as the on-site interaction *U* in FHM, we propose generating new configurations using the time evolution operator $Û\left(x_{e},\tau \right)=\exp\left(-iĤ\left(x_{e}\right)\tau \right)$ , where *x*_*e*_ may differ from *x* to optimize sampling efficiency. For real Hamiltonians considered in this work, the Hermiticity of ensures that it is also symmetric, such that eq 14 still holds. We will refer to this proposal as the Quantum proposal in the following context. Very recently, ref 59 proposed another way to combine VMC and QeMCMC, where a surrogate network based on the classical Ising model is introduced to first fit the target distribution. The QeMCMC^51^ algorithm is then directly applied to sample the probability distribution of the surrogate network, and the energy in VMC is estimated using a reweighting technique. In contrast, our approach is much simpler since it does not require the fitting procedure. However, in our case, a good effective Hamiltonian $Ĥ\left(x_{e}\right)$ driving the unitary evolution needs to be designed for efficient sampling.

**Figure 1:**
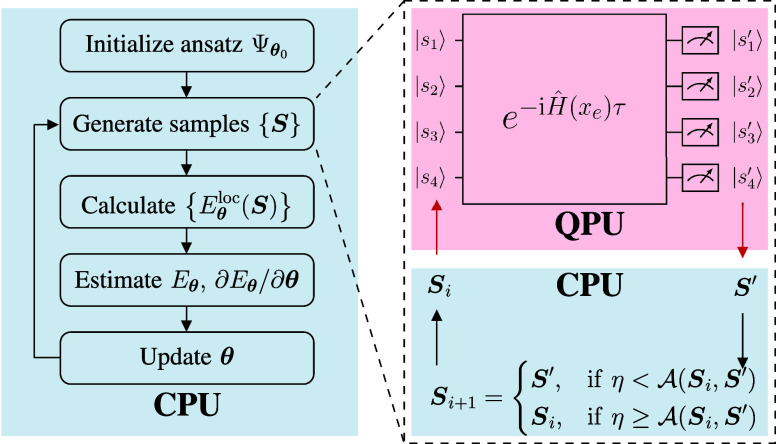
Flowchart of the QA-VMC algorithm. The red box highlights the quantum step executed on quantum processor units (QPU), where a quantum time-evolution governed by a chosen Hamiltonian *Ĥ* satisfying ${Ĥ}^{T}=Ĥ$ and measurements are employed to propose new configurations. All other parts of the algorithm are executed on classical computers. The acceptance probability $A\left(S_{i},S_{j}\right)$ is determined by eq 14, and η ∈ [0, 1] is a uniformly distributed random number.

To gain a deeper understanding of the Quantum proposal, we decompose the corresponding proposal probability $Q^{q}\left(S_{i},S_{j};x_{e},\tau \right)$ into two parts

15 $$\begin{array}{ll} Q^{q}\left(S_{i},S_{j};x_{e},\tau \right) & =|\langle S_{j}|\exp\left(-iĤ\left(x_{e}\right)\tau \right){|S_{i}\rangle |}^{2}\\ & =\underset{n}{\sum }p_{n}\left(S_{i};x_{e}\right)p_{n}\left(S_{j};x_{e}\right)+\Omega \left(S_{i},S_{j};x_{e},\tau \right) \end{array}$$

where *p*_*n*_(***S***_*i*_; *x*_*e*_) = |⟨ ***S***_*i*_|Ψ_*n*_⟩|^2^ and {|Ψ_*n*_⟩} represents the eigenstates of $Ĥ\left(x_{e}\right)$ , and Ω­(***S***_*i*_, ***S***_*j*_; *x*_*e*_, τ) is given by

$$\Omega \left(S_{i},S_{j};x_{e},\tau \right)=2R\underset{n>m}{\sum }\langle S_{j}|\Psi_{n}\rangle \langle \Psi_{n}|S_{i}\rangle e^{i(E_{m}-E_{n})\tau }$$ 16

The first term in eq 15 is time-independent and will be referred to as the Effective proposal

17 $$Q^{eff}\left(S_{i},S_{j};x_{e}\right)=\underset{n}{\sum }p_{n}\left(S_{i};x_{e}\right)p_{n}\left(S_{j};x_{e}\right)$$

since it can be verified that $\underset{S_{j}}{\sum }Q^{eff}\left(S_{i},S_{j};x_{e}\right)=1$ . While $Q^{eff}\left(S_{i},S_{j};x_{e}\right)$ is inefficient to implement on classical computers and quantum computers directly, it provides valuable insights into the usefulness of the Quantum proposal based on the following observations:

First, for a Hamiltonian $Ĥ\left(x_{e}\right)$ without degeneracy, the time-averaged $Q^{q}$ over τ ∈ (−*∞*, + *∞*) equals $Q^{eff}$ , i.e.

18 $$Q^{eff}\left(S_{i},S_{j};x_{e}\right)=\underset{\tau \to +\infty }{\lim}\frac{1}{2\tau }\int_{-\tau }^{+\tau }Q^{q}\left(S_{i},S_{j};x_{e},\tau^{\prime }\right)d\tau^{\prime }$$

This implies that if we randomly select τ within some sufficiently large interval (−*T*, + *T*), the averaged $Q^{q}$ will equal $Q^{eff}$ . This point is further illustrated in Supporting Information for different model systems.

Second, the proposed move using $Q^{eff}$ has a more intuitive interpretation, because eq 17 can be understood as follows: given a configuration ***S***_*i*_, first randomly select an eigenstate |Ψ_*n*_⟩ according to the conditional probability distribution *P*(*n*| ***S***_*i*_) ≡ *p*_*n*_(***S***_*i*_; *x*_*e*_), and then randomly select a configuration ***S***_*j*_ based on the conditional probability distribution *P*(***S***_*j*_|*n*) ≡ *p*_*n*_(***S***_*j*_; *x*_*e*_). Thus, if *p*_0_(***S***_*i*_) and *p*_0_(***S***_*j*_) for the ground state are both large, *Q* ^eff^(***S***_*i*_, ***S***_*j*_; *x*_*e*_) will also be large, regardless of the Hamming distance between ***S***_*i*_ and ***S***_*j*_. This suggests that for a ground state probability distribution concentrated on some configurations with large Hamming distances the Effective proposal can offer a significant advantage over classical proposals. Based on eq 18, we expect the Quantum proposal to exhibit similar behavior.

A primary objective of this work is to examine whether the QA-VMC algorithm can potentially enhance the convergence of MCMC simulations, thereby providing computational efficiency gains for VMC. To investigate this, we apply this algorithm to FHMs and molecular systems across various parameter ranges and system sizes. Through a comprehensive comparative analysis with conventional classical proposals, we evaluate the performance of QA-VMC from multiple perspectives, as detailed in the following section.

### Figures of Merit

#### Absolute Spectral Gap

The convergence rate of the Markov chain can be quantitatively characterized by its mixing time^27,51^ *t*_mix_(ε), which is the minimum number of steps *t* required for the Markov chain to converge to its stationary distribution within a predefined tolerance threshold *ε*, i.e.,

19 $$t_{mix}\left(\varepsilon \right):=\min\left\{t:\max_{S_{i}}{∥P^{t}\left(S_{i},\cdot \right)-\pi \left(\cdot \right)∥}_{TV}\leq \varepsilon \right\}$$

where ∥·∥_TV_ denotes the total variation distance,^27^ quantifying the discrepancy between the chain’s distribution after *t* steps and the stationary distribution. While the exact computation of *t*_mix_(ε) is generally intractable, it can be effectively bounded by the absolute spectral gap δ via^27^

20 $$\left(\delta^{-1}-1\right)\;\ln\left(\frac{1}{2\varepsilon }\right)\leq t_{mix}\left(\varepsilon \right)\leq \delta^{-1}\;\ln\left(\frac{1}{\varepsilon \;\min_{S}\pi \left(S\right)}\right)$$

Here, δ = 1 – |λ_2_| ∈ [0, 1] is the difference between the absolute values of the two largest eigenvalues (λ_1_ = 1 and λ_2_) of the transition matrix P ([Disp-formula eq9]), which can be computed through matrix diagonalization, making δ more readily accessible than the mixing time. As is evident from eq 20, the spectral gap δ exhibits an inverse relationship with the bounds of the mixing time, thereby serving as a precise quantitative measure for assessing Markov chain convergence.^51^ Specifically, a larger spectral gap δ implies a smaller *t*_mix_(ε) and hence faster convergence to the stationary distribution. However, it is crucial to acknowledge that the practical computation of δ is limited by the exponential growth of Hilbert space. Therefore, in this work we employ an extrapolation approach adopted in the QeMCMC work^51^ to establish a relationship between δ and system size *N* obtained from computationally feasible systems. This enables us to estimate the asymptotic behavior of δ for larger systems that are not feasible for diagonalization.

#### Autocorrelation Time

Apart from the absolute spectral gap, autocorrelation time is another valuable metric for assessing the convergence of MCMC algorithms.^67^ This metric is widely used in practice, because it directly captures the convergence behavior of the Markov chain, particularly in terms of how long the chain retains memory of its previous states. For a given operator *Ô*, the integrated autocorrelation time τ_*O*_ is defined as

21 $$\tau_{O}=1+2\overset{\infty }{\underset{\tau =1}{\sum }}\rho_{O}\left(\tau \right)\;,\rho_{O}\left(\tau \right)=\frac{c_{O}\left(\tau \right)}{c_{O}\left(0\right)}$$

where *c*_*O*_(τ) represents the autocovariance function at lag τ

22 $$c_{O}\left(\tau \right)=\frac{\overset{N_{s}-\tau }{\underset{i=1}{\sum }}\left(O_{loc}\left(S_{i}\right)-\mu_{O}\right)\left(O_{loc}\left(S_{i+\tau }\right)-\mu_{O}\right)}{N_{s}-\tau }$$

Here, $O_{loc}\left(S_{i}\right)\equiv \frac{\left\langle S_{i}\left|Ô\right|\Psi \right\rangle }{\langle S_{i}|\Psi }$ , $\mu_{O}=\frac{1}{N_{s}}\overset{N_{s}}{\underset{i=1}{\sum }}O_{loc}\left(S_{i}\right)$ denotes the sample average, and *N*_*s*_ represents the sample size. A smaller τ_*O*_ indicates faster convergence of the estimator to its mean, reflecting the efficient mixing of the chain. Conversely, a larger τ_*O*_ value suggests strong correlations among samples and slow mixing. The integrated autocorrelation time is related to the effective sample size, *N*_eff_ by *N*_eff_ = *N*_*s*_/τ_*O*_. Thus, it can serve as a practical and intuitive measure of the chain’s convergence properties. We used the algorithm introduced in ref 67 to estimate τ_*O*_.

#### Metric for Potential Quantum Speedup

To explore the potential quantum speedup of the Quantum proposal compared to classical proposals, we analyze the asymptotic behavior of the quantity $T_{eff}=\delta^{-1}t_{s}$ , which will be referred to as the effective runtime. Here, δ^–1^ estimates the number of steps required to reach equilibrium, and *t*_*s*_ is the runtime of a single execution of a classical or quantum move. Thus, $T_{eff}$ roughly estimates the runtime of an ideal MCMC algorithm. The spectral gap δ can be modeled by an exponential function with respect to the system size *N* via δ­(*N*) = *a*2^–*kN*^.^51^ Then, the ratio between the effective runtime of a classical proposal $T_{eff,c}$ and that of the Quantum proposal $T_{eff,q}$ proposals can be expressed as

23 $$\frac{T_{eff,c}}{T_{eff,q}}=\frac{\delta_{c}^{-1}t_{s,c}}{\delta_{q}^{-1}t_{s,q}}=\frac{a_{q}t_{s,c}}{a_{c}t_{s,q}}2^{\left(k_{c}-k_{q}\right)N}$$

The runtime *t*_*s*,*c*_ for classical moves considered in this work scales at most polynomially with the system size *N*. Consequently, if the runtime *t*_*s*,*q*_ for the quantum case also scales polynomially, then $T_{eff,c}>T_{eff,q}$ for sufficiently large systems, provided that *k*_*c*_ > *k*_*q*_. However, if *t*_*s*,*q*_ scales exponentially as *O*(2^*bN*^), a potential speedup can exist only if *k*_*c*_ > *k*_*q*_ + *b*. Therefore, in addition to the asymptotic behavior of δ characterized by the exponent *k*, the potential quantum advantage is also critically dependent on the scaling of *t*_*s*,*q*_ with respect to that of *N*. In the following sections, we focus on the asymptotic behaviors of both δ and *t*_*s*,*q*_.

## Results and Discussion

### Fermi–Hubbard Model

We begin by evaluating the performance of the QA-VMC algorithm for the FHM,^68^ which serves as a benchmark for both classical and quantum variational methods.^69,70^ The Hamiltonian of the FHM is given by

24 $$Ĥ\left(U\right)=-t\underset{\left\langle i,j\right\rangle }{\sum }\underset{\sigma }{\sum }\left({â}_{i\sigma }^{†}{â}_{j\sigma }+\mathrm{h.c.}\right)+U\underset{i}{\sum }{\mathrm{n̂}}_{i\alpha }{\mathrm{n̂}}_{i\beta }$$

where the hopping parameter *t* = 1, *U* is the on-site interaction, σ ∈ {α, β}, ${â}_{q}^{\left(†\right)}$ represents Fermionic annihilation (creation) operators, and ⟨*i*, *j*⟩ represents the summation over nearest-neighbor sites. Additionally, we use the Jordan–Wigner mapping^71^ to transform the Fermionic Hamiltonian *Ĥ* into a qubit Hamiltonian expressed as a linear combination of Pauli terms, i.e. $Ĥ=\underset{k}{\sum }h_{k}P_{k}$ with *P*_*k*_ ∈ {*I*,*X*,*Y*,*Z*}^⊗*N*^, and the occupation number vectors into corresponding qubit configurations. In this study, we focus on the ground state of the FHM with open boundary conditions (OBC) at half-filling. In addition to the aforementioned classical proposals, we also extend the ExcitationSD proposal by incorporating a global spin flip operation, denoted by ExcitationSD+flip. In this proposal, with equal probability, either a random single/double excitation or a global spin flip is performed.

We first analyze the asymptotic behavior for the absolute spectral gaps with the system size *N* and the on-site interaction *U* for the exact ground state of the one-dimensional (1D) FHM. For the Quantum proposal, δ is a function of evolution time τ. As shown in the Supporting Information, as τ increases, δ first reaches that of the Effective proposal, denoted by δ_eff_, and then oscillates around it. To examine the best performance that the Quantum proposal can achieve, we take the maximal absolute spectral gap by scanning τ from 0.1 to 20 with a step size of 0.2 for each *U* and *N*. The results obtained with different proposals are summarized in Figure 2, where we also plot the results obtained by the Quantum proposal with a fixed *U*_*e*_ = 8 for all *U*. Figure 2a indicates that the Quantum (*U*_*e*_ = *U*) proposal and that with a fixed *U*_*e*_ = 8 generally exhibit larger spectral gaps δ than classical proposals for *U* ∈ [1, 16] and behave similarly to the corresponding Effective proposals. Notably, around *U* = 8, δ of the Quantum (*U*_*e*_ = *U*) proposal is approximately an order of magnitude larger than that of the ExcitationSD proposal in the 10-site 1D FHM. However, as *U* increases to infinity, while the absolute spectral gaps of the ExcitationSD, ExcitationSD+flip, and Uniform proposals approach a fixed value, those of the Quantum (*U*_*e*_ = *U*), Effective (*U*_*e*_ = *U*), and Exchange proposals decrease. This is because in the *U* = *∞* limit Markov chains generated by these proposals become reducible. Using a fixed *U*_*e*_ = 8 in the Quantum proposal can avoid this problem, leading to a steady δ over a wider range of *U*.

**Figure 2:**
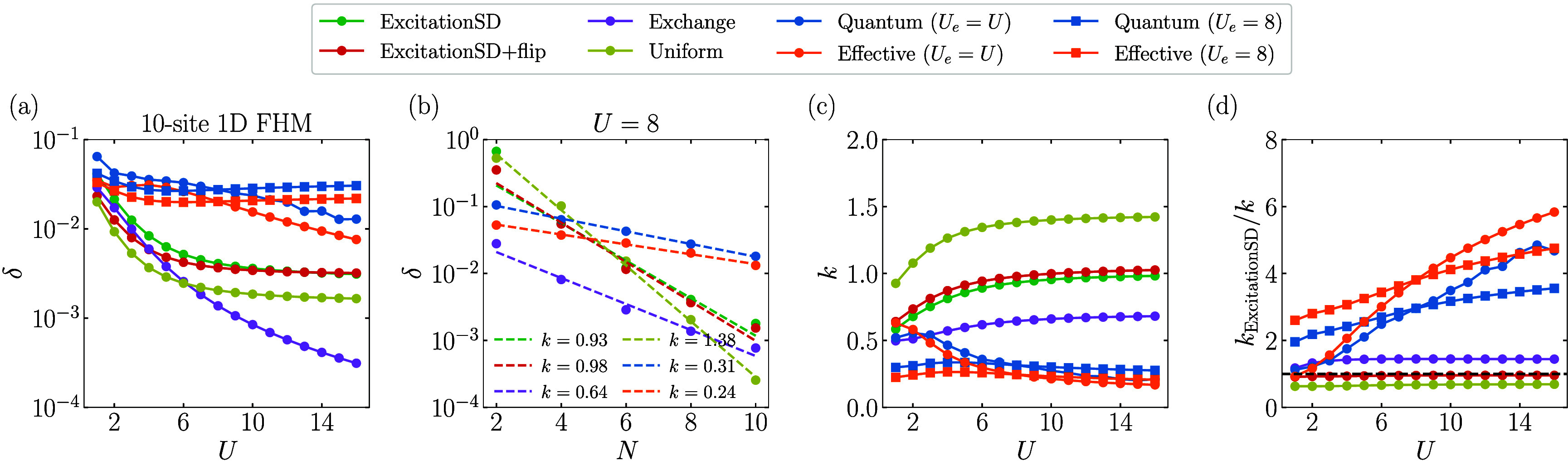
Absolute spectral gap δ obtained by diagonalizing the transition matrix P of each proposal for the ground state of the 1D FHM. For the Quantum proposal, δ is obtained as the maximal absolute spectral gap by scanning τ from 0.1 to 20 with a step size of 0.2. (a) Illustration for δ of different proposals as a function of *U* for the 10-site 1D FHM. (b) δ of different proposals as a function of the system size *N* for *U* = 8. The function δ ≈ *a*2^–*kN*^ is used to fit the data of each proposal, and the dashed lines are the fitted curves with the obtained *k* shown in the inset. (c) The fitted exponent *k* as a function of *U*. (d) *k*_rel_ = *k*_ExcitationSD_/*k* as a function of the parameter *U*. The black dashed line represents *k*_rel_ = 1.0.

Figure 2b demonstrates that δ for a fixed value of *U* exhibits an exponential decay with increasing system size *N* for all proposals. Following the approach outlined in ref 51, we fit the data using δ­(*N*) = *a*2^–*kN*^. Note that both prefactor *a* and exponent *k* depend on *U*. The Quantum (*U*_*e*_ = *U*) and Effective (*U*_*e*_ = *U*) proposals are found to have the smallest exponents at *U* = 8. Figure 2c presents the obtained exponents *k* for different *U* using the same fitting procedure, and Figure 2d illustrates the relative performance by plotting the ratio *k*_ExcitationSD_/*k*. We find that for small *U* (≈1), the Quantum (*U*_*e*_ = *U*) proposal does not provide an advantage over classical proposals. However, it does exhibit an advantage for a larger *U*, indicating the potential for quantum speedup. In comparison, the Quantum approach with a fixed *U*_*e*_ = 8 shows a more balanced performance across all *U* values. As shown in the Supporting Information, the advantage of the Quantum proposal in the exponent over classical proposals persists for 2D and random FHMs.

To understand how the Quantum proposal speeds up the convergence of the MCMC sampling at larger *U*, we introduce the configuration “energy” defined by

25 $$\epsilon \left(S\right)=-\log_{10}P\left(S\right),P\left(S\right)=|\langle S{|\Psi_{0}\rangle |}^{2}$$

which is analogous to the energy function in the classical Boltzmann distribution. Specifically, a configuration with high energy ϵ­(***S***) corresponds to a low probability *P*(***S***), and a large increase in energy

26 $$\Delta \epsilon =\epsilon \left(S_{j}\right)-\epsilon \left(S_{i}\right)=\log_{10}\left(P\left(S_{i}\right)/P\left(S_{j}\right)\right)$$

will lead to a low acceptance rate in MCMC sampling. In Figure 3, we plot the two-dimensional histogram of different proposal probabilities $Q\left(S_{i},\cdot \right)$ for the 10-site 1D FHM with *U* = 8, with the Hamming distance and “energy” change △ϵ as the *x* and *y* axes, respectively. Here, the qubit configuration ***S***_*i*_ = (−1, 1, 1, −1, ..., −1, 1, 1, −1) is one of the two configurations with the largest ground-state probability (see the Supporting Information). Its spin-flipped counterpart (1, −1, −1, 1, ..., 1, −1, −1, 1) has an identical probability due to spin-flip symmetry ( $\left[Ĥ,{Û}_{SF}\right]=0$ , where ${Û}_{SF}=e^{i\pi \left({Ŝ}_{x}-\mathrm{N̂}/2\right)}$ ), but the largest Hamming distance (=20) from ***S***_*i*_. As shown in Figure 3a–c, the ExcitationSD, ExcitationSD+flip, and Exchange proposals generate configurations that move only by specific Hamming distances. Moreover, the newly generated configurations often exhibit a significant increase in “energy”, leading to a reduced acceptance rate in MCMC sampling. Figure 3d shows that although the Uniform proposal allows transitions over unrestricted Hamming distances, it predominantly generates high-energy configurations, thereby also decreasing the MCMC acceptance rate. In contrast, Figure 3e,f demonstrates that the Quantum and Effective proposals can generate configurations with a range of Hamming distances while maintaining relatively low “energy”. This distinctive property significantly enhances Markov chain convergence, differentiating quantum moves from classical moves.

**Figure 3:**
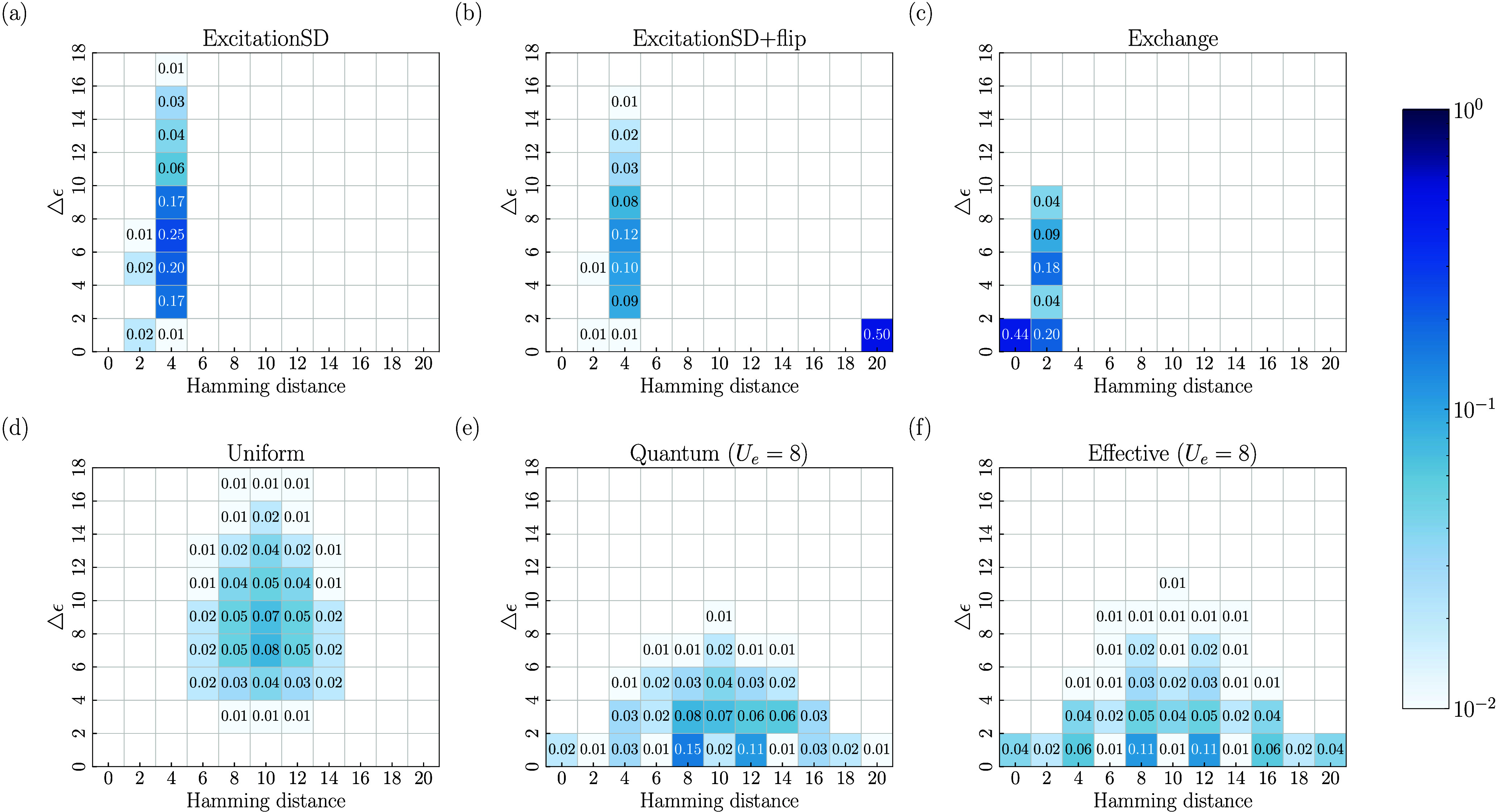
Comparison of different proposal probabilities $Q\left(S_{i},\cdot \right)$ from the qubit configuration ***S***_*i*_ = (−1, 1, 1, – 1, ..., – 1, 1, 1, – 1) with the largest ground-state probability in the 10-site 1D FHM with *U* = 8. (a–f) Two-dimensional histograms of $Q\left(S_{i},\cdot \right)$ with the Hamming distance (between ***S***_*j*_ and ***S***_*i*_) and the “energy” gap (△ϵ = log_10_(*P*(***S***_*i*_)/*P*(***S***_*j*_)) as the *x* and *y* axes, respectively: (a) ExcitationSD; (b) ExcitationSD+flip; (c) Exchange; (d) Uniform; (e) Quantum (*U*_*e*_ = 8).; (f) Effective (*U*_*e*_ = 8).

As discussed in the previous section, it is also crucial to examine the asymptotic behavior of runtime *t*_*s*,*q*_ in order to assess whether the Quantum proposal can achieve a quantum advantage in computational time. The runtime *t*_*s*,*q*_ of a single quantum move is proportional to evolution time τ. Here, we consider the evolution time required to first reach a certain fraction of δ_eff_ and analyze its dependence on the system size. This is motivated by the observation that as the evolution time increases, the spectral gap of the Quantum proposal oscillates around δ_eff_ (see the Supporting Information for details). Figure 4 shows the evolution time τ at which δ of the Quantum proposals (*U*_*e*_ = *U* and *U*_*e*_ = 8) first exceeds *cδ*_eff_ for *c* = 0.6, 0.7, and 0.8, respectively. Notably, the required evolution time does not increase rapidly with the system size. In particular, it reaches a plateau for both *U* = 4 and *U* = 8. Similar behaviors are also observed for 2D FHMs shown in the Supporting Information. Based on eq 23, these findings suggest that the Quantum proposal, with an appropriately chosen parameter *U*_*e*_, may offer a potential quantum speedup over classical proposals for sufficiently large systems.

**Figure 4:**
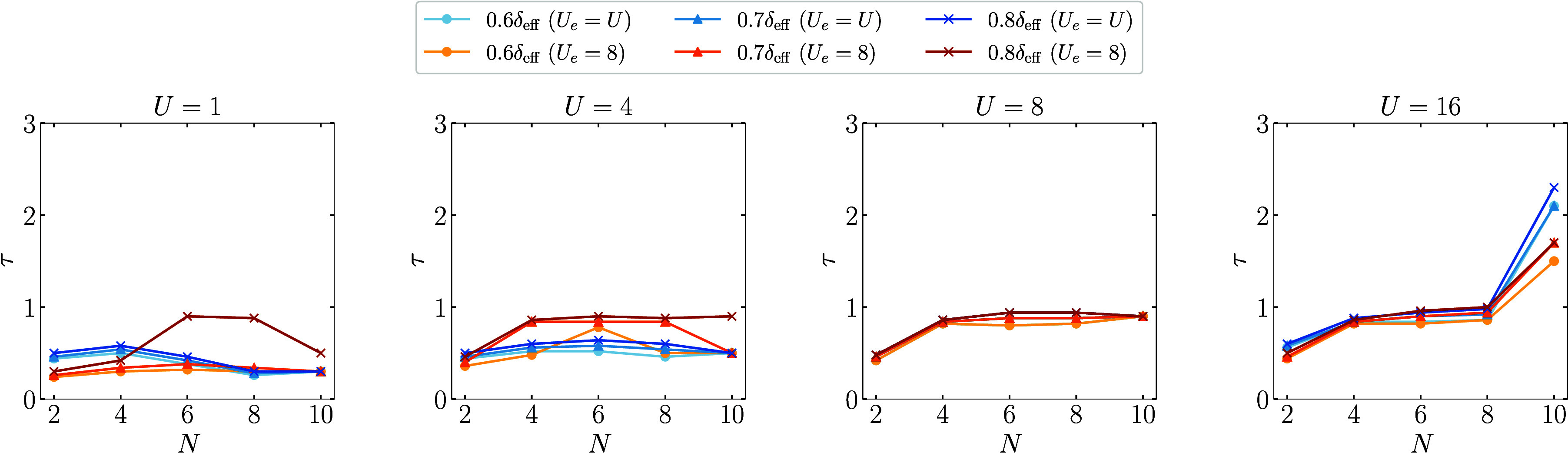
Evolution time τ required for δ of the Quantum proposal to first exceed *cδ* ^eff^ (*c* = 0.6, 0.7, and 0.8) as a function of the system size *N* for the ground state of 1D FHMs with different *U*.

To further assess the quality of samples generated by different proposals, we evaluate an observable $\left\langle {\mathrm{n̂}}_{1\alpha }{\mathrm{n̂}}_{N\beta }\right\rangle$ using the MCMC algorithm for the exact ground state of the 10-site 1D FHM with *U* = 8. Figure 5a presents the results of 100 independent Markov chains for each proposal. The Quantum proposal demonstrates superior performance, yielding more accurate results with smaller variations for a given sample size of *N*_*s*_. Compared to the best classical proposal (ExcitationSD+flip) for this observable, the Quantum proposal reduces the maximum error and standard deviation by approximately a factor of 3 for *N*_*s*_ = 10^5^, as shown in Figure 5b,c. This improvement suggests that the effective sample size *N*_eff_ is roughly 9 times larger, which aligns well with the estimated integrated autocorrelation time $\tau_{n_{1\alpha }n_{N\beta }}$ for *N* = 10 depicted in Figure 5d. We extend the same analysis to other system sizes and fit the obtained $\tau_{n_{1\alpha }n_{N\beta }}$ as a function of *N* using *a*2^*kN*^ in Figure 5d. The results reveal that the Quantum proposal exhibits the smallest *k*, and hence the slowest increase in $\tau_{n_{1\alpha }n_{N\beta }}$ as the system size *N* increases, which is consistent with the trend observed for the absolute spectral gap. This further underscores the higher quality of the samples produced by the Quantum proposal.

**Figure 5:**
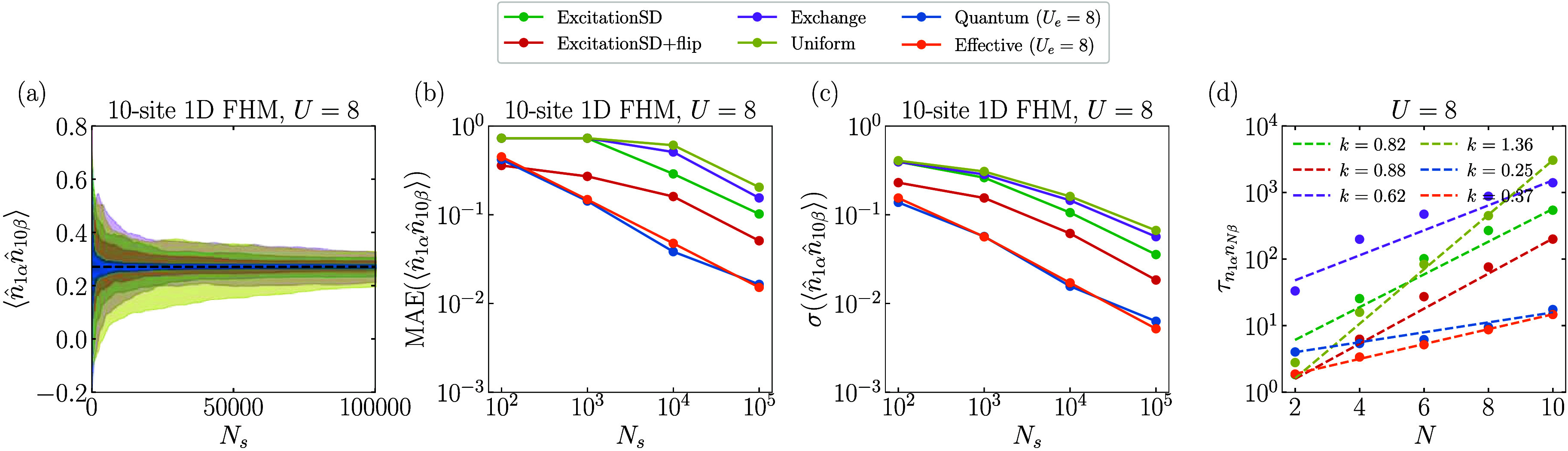
Estimation of an observable $\left\langle {\mathrm{n̂}}_{1\alpha }{\mathrm{n̂}}_{N\beta }\right\rangle$ by 100 independent Markov chains with different proposals for the exact ground state of 1D FHM with *U* = 8. (a) The distribution of the estimated $\left\langle {\mathrm{n̂}}_{1\alpha }{\mathrm{n̂}}_{10\beta }\right\rangle$ for a given *N*_*s*_ with different proposals. The black dashed line represents the exact value. (b) maximum absolute errors (MAE) for the estimated $\left\langle {\mathrm{n̂}}_{1\alpha }{\mathrm{n̂}}_{10\beta }\right\rangle$ as a function of *N*_*s*_. (c) standard deviation σ for the estimated $\left\langle {\mathrm{n̂}}_{1\alpha }{\mathrm{n̂}}_{10\beta }\right\rangle$ as a function of *N*_*s*_. (d) Estimated $\tau_{n_{1\alpha }n_{10\beta }}$ as a function of *N* for different proposals using the MCMC algorithm with *N*_*s*_ = 10^7^. The data were further fitted using *a*2^*kN*^ (dashed lines) with the obtained exponents shown in the inset.

Finally, we illustrate the performance of the QA-VMC algorithm in practical applications by combining it with the RBM ansatz (α = 3) to target the ground-state of the 10-site 1D FHM with *U* = 8. The results obtained using two different sample sizes (*N*_*s*_ = 10^4^ and *N*_*s*_ = 10^5^) are presented in Figure 6. Figure 6a,b demonstrates that the variational energy computed by QA-VMC converges more efficiently toward the exact ground-state energy, requiring fewer samples *N*_*s*_ compared with classical proposals. Specifically, VMC with classical proposals fails to converge to the correct ground state using *N*_*s*_ = 10^4^. In contrast, the convergence trajectory of QA-VMC aligns more closely with the optimization using the exact gradients (black lines), highlighting its superior efficiency due to the higher quality of samples. Additionally, Figure 6c,d displays the estimated $\left\langle {\mathrm{n̂}}_{1\alpha }{\mathrm{n̂}}_{10\beta }\right\rangle$ during the VMC optimizations. The results obtained with the Quantum proposals are found to exhibit better accuracy and smaller oscillations at the same sample size *N*_*s*_ compared with the classical proposals. This shows the potential of QA-VMC for significantly enhancing the performance of the VMC algorithm for large systems.

**Figure 6:**
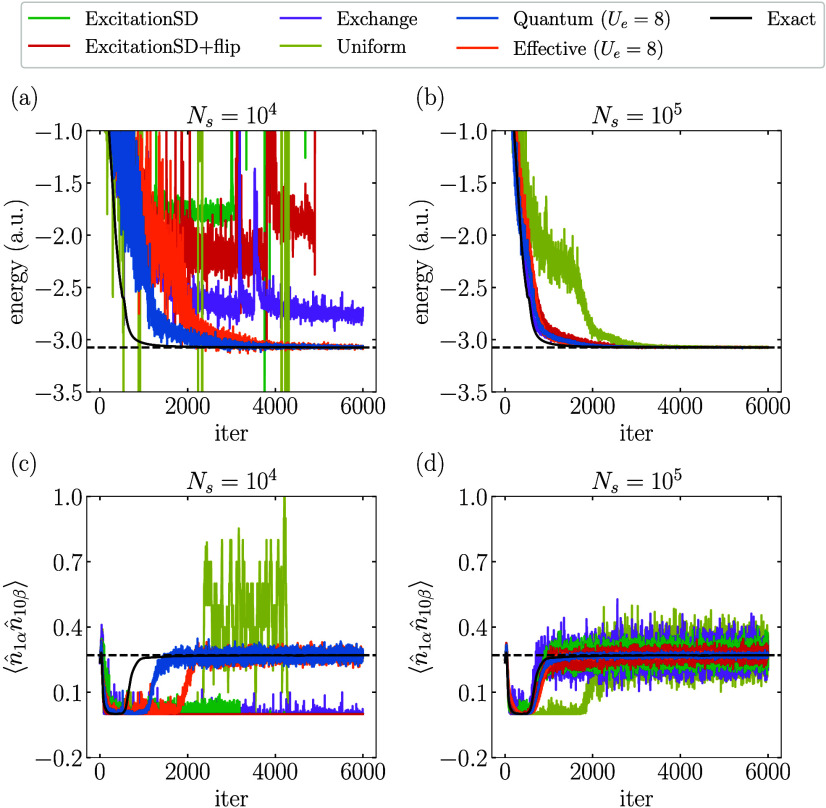
VMC optimization process of different proposals using the RBM (α = 3) ansatz for 10-site 1D FHM with *U* = 8: (a,b) energy; (c,d) $\left\langle {\mathrm{n̂}}_{1\alpha }{\mathrm{n̂}}_{10\beta }\right\rangle$ . Black solid lines in (a) and (b) represent the optimization trajectory using the exact gradients without sampling. Black dashed lines represent the exact ground-state energy in (a) and (b) or $\left\langle {\mathrm{n̂}}_{1\alpha }{\mathrm{n̂}}_{10\beta }\right\rangle$ for the exact ground state in (c) and (d).

### Molecules

After benchmarking QA-VMC for FHMs across various system sizes and interaction parameters, we applied it to chemical systems with more realistic interactions. The molecular Hamiltonian is given by

27 $$Ĥ=\underset{pq,\sigma }{\sum }h_{pq}{â}_{p\sigma }^{†}{â}_{q\sigma }+\frac{1}{2}\underset{pqrs,\sigma \tau }{\sum }g_{pqrs}{â}_{p\sigma }^{†}{â}_{r\tau }^{†}{â}_{s\tau }{â}_{q\sigma }+E_{nuc}$$

where *h*_*pq*_ and *g*_*pqrs*_ denote the one- and two-electron molecular integrals, respectively, and *E*_nuc_ represents the nuclear repulsion energy. The Fermionic Hamiltonian is then transformed into a qubit Hamiltonian via the Jordan–Wigner mapping^71^ for subsequent studies. Analogous to the effective Hamiltonian approach employed in the Hubbard model, we can construct an effective Hamiltonian for molecular systems by fixing the bond length *R*_*e*_, and the resulting proposal will be defined by the Quantum (*R*_*e*_) proposal.

Additionally, we introduce another way to design an effective Hamiltonian, by incorporating an artificial hopping term into the Hamiltonian, viz.

28 $$Ĥ\left(\gamma_{e}\right)=\left(\left(1-\gamma_{e}\right)\underset{pq,\sigma }{\sum }h_{pq}{â}_{p\sigma }^{†}{â}_{q\sigma }+\gamma_{e}\alpha {Ĥ}_{hopping}\right)+\frac{1}{2}\underset{pqrs,\sigma \tau }{\sum }g_{pqrs}{â}_{p\sigma }^{†}{â}_{r\tau }^{†}{â}_{s\tau }{â}_{q\sigma }+E_{nuc}$$

where ${Ĥ}_{hopping}$ is

29 $${Ĥ}_{hopping}=-\underset{p\neq q,\sigma }{\sum }{â}_{p\sigma }^{†}{â}_{q\sigma }$$

Here, γ_*e*_ ∈ [0.0, 1.0] is a tunable parameter that governs the relative contribution of the hopping term in the one-body part, and the normalization factor $\alpha =\frac{{∥h∥}_{F}}{\sqrt{n\left(n-1\right)}}$ , where *n* is the number of spatial orbitals, ensures appropriate scaling of the one-electron component. We will denote the Quantum proposal using eq 28 by the Quantum (hopping, γ_*e*_) proposal. When γ_*e*_ = 0.0, the Quantum (hopping, γ_*e*_ = 0.0) proposal reduces to the Quantum (*R*_*e*_ = *R*) proposal. Conversely, setting γ_*e*_ = 1.0 replaces the entire one-body term with the hopping operator. As the optimal value of γ_*e*_ is generally unknown, we adopt a stochastic strategy as in ref 51, in which γ_*e*_ is sampled from a uniform distribution in the interval [0.1,0.4]. The resulting proposal will be denoted by the Quantum (hopping, random) proposal (see the Supporting Information for details of implementation).

#### Hydrogen Chains

A typical example, closely related to FHMs, is the hydrogen chains at varying interatomic distances *R*, which can undergo transitions from weakly correlated systems at small *R* to strongly correlated systems at larger *R*. We employed orthonormalized atomic orbitals (OAO) obtained with the STO-3G basis. Figure 7 presents the absolute spectral gaps δ obtained with different proposals for the ground state of hydrogen chains H_*n*_. As depicted in Figure 7a, as the bond length *R* increases from 0.5 to 2.5 Å, the absolute spectral gap δ for the Quantum (*R*_*e*_ = *R*) proposal is generally much greater than those of classical proposals. Similar to FHMs in the large *U* limit, δ for the Exchange, Quantum (*R*_*e*_ = *R*), and Effective (*R*_*e*_ = *R*) proposals decreases to zero as *R* increases, due to the lost of irreducibility for the generated Markov chains in the *R* = *∞* limit. In contrast, other proposals maintain a nonzero δ at a large *R*. In particular, by fixing *R*_*e*_ to a specific value, such as 2.0 Å, the spectral gap of the Quantum proposal can sustain a large value across different *R* (see Figure 7a). Figure 7b shows that δ decays exponentially with system size and is well-fitted by the function *a*2^–*kN*^. At *R* = 2.0 Å, the fitted exponent *k* for the Quantum proposal is only about one-third of that of the widely used ExcitationSD proposal, indicating a significant potential speedup for large systems. Figure 7c,d display the fitted exponents *k* for different bond lengths and the relative exponents *k*_rel_ = *k*_ExcitationSD_/*k* compared against that of ExcitationSD, respectively. It is evident that at larger *R* > 1.5 Å, where the ground-state configurations become more concentrated on some configurations separated by large Hamming distances (see the Supporting Information), the Quantum proposals start to outperform classical proposals.

**Figure 7:**
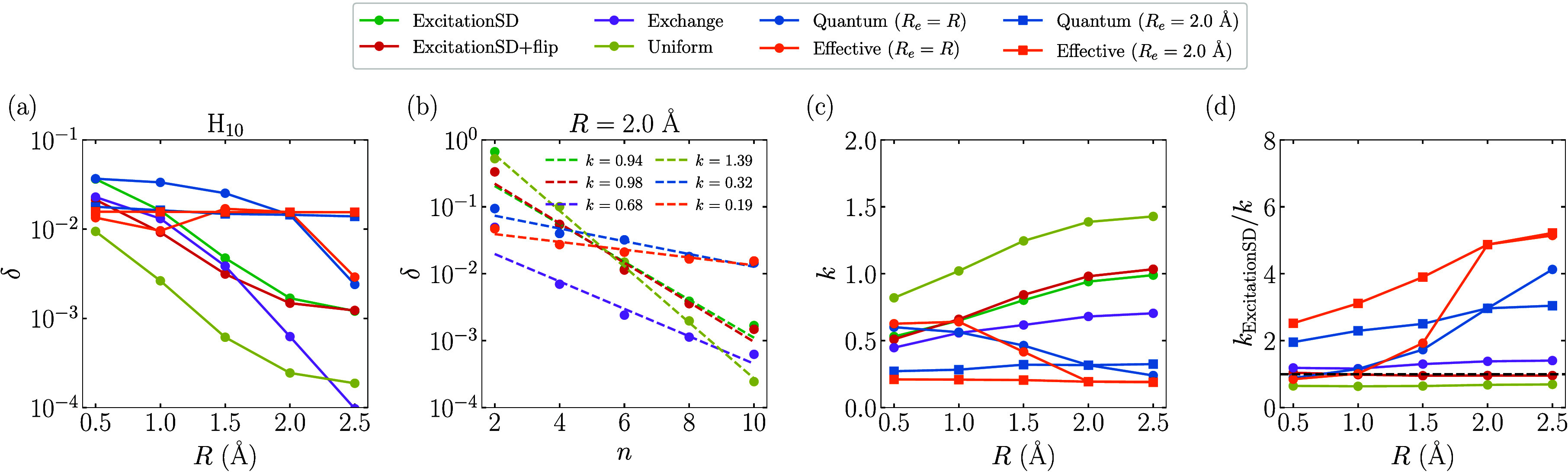
Absolute spectral gap δ obtained by diagonalizing the transition matrix P of each proposal for the ground state of the hydrogen chains H_*n*_. For the Quantum proposal, δ is obtained as the maximal absolute spectral gap by scanning τ from 0.1 to 60.0 with a step size of 0.2. (a) δ of different proposals as a function of *R* for H_10_. (b) δ of different proposals as a function of the system size *n* at *R* = 2.0 Å. The function *a*2^–*kN*^ is used to fit the data of each proposal, and the dashed lines are the fitted curves with the obtained *k* shown in the inset. (c) The fitted exponent *k* as a function of *R*. (d) *k*_rel_ = *k*_ExcitationSD_/*k* as a function of the parameter *U*. The black dashed line represents *k*_rel_ = 1.0.

Figure 8 presents the absolute spectral gaps δ obtained from both the Quantum and Effective proposals, using the effective Hamiltonian $Ĥ\left(\gamma_{e}\right)$ defined in eq 28. In Figure 8a, the Quantum (hopping) proposals exhibit robust performance with the bond length *R* from 0.5 to 2.5 Å. In particular, in contrast to the quantum (*R*_*e*_ = *R*) proposal, the absolute spectral gap δ for the Quantum (hopping) proposals remains nonvanishing at large bond lengths, indicating improved performance in the dissociation limit. Figure 8b shows that a small value of γ_*e*_ results in a favorable scaling factor *k* at *R* = 2.0 Å. Additionally, the Quantum (hopping, random) proposal achieves a similarly small *k*, highlighting the effectiveness of the randomized strategy. A more detailed comparison of the scaling behavior is shown in Figure 8c, where a clear hierarchy emerges: *k* (γ_*e*_ = 0.1) < *k* (random) < *k* (γ_*e*_ = 0.9). Finally, Figure 8d demonstrates that the Quantum (hopping, random) proposal consistently outperforms classical approaches for bond lengths of >1.5 Å, underscoring its advantage in strongly correlated regimes.

**Figure 8:**
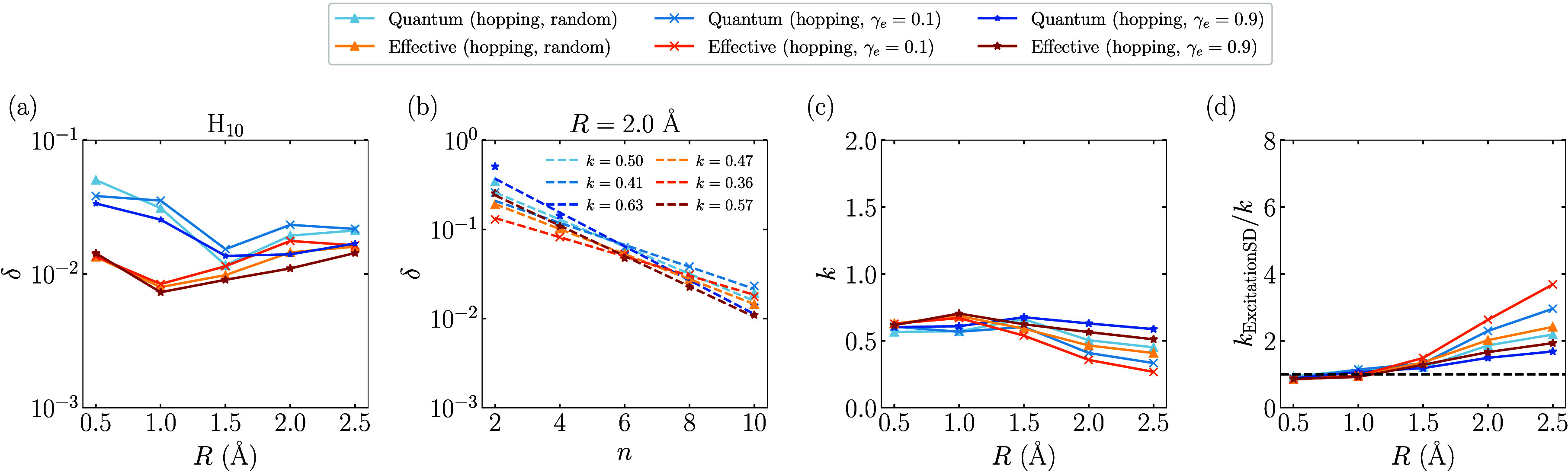
Absolute spectral gap δ obtained by diagonalizing the transition matrix P of different Quantum (hopping) proposals for the ground state of the hydrogen chains H_*n*_. For different Quantum (hopping) proposals, δ is obtained as the maximal absolute spectral gap by scanning τ from 0.1 to 60.0 with a step size of 0.2. (a) δ of different Quantum (hopping) proposals as a function of *R* for H_10_. (b) δ of different Quantum (hopping) proposals as a function of the system size *n* at *R* = 2.0 Å. The function *a*2^–*kN*^ is used to fit the data of each proposal, and the dashed lines are the fitted curves with the obtained *k* shown in the inset. (c) The fitted exponent *k* as a function of *R*. (d) *k*_rel_ = *k*_ExcitationSD_/*k* as a function of the parameter *U*. The black dashed line represents *k*_rel_ = 1.0.

Finally, we illustrate the performance of QA-VMC combined with the RBM ansatz (α = 3) for computing the ground state of the hydrogen chain H_10_ and the observable $\left\langle {\mathrm{n̂}}_{1\alpha }{\mathrm{n̂}}_{10\beta }\right\rangle$ at *R* = 2.0 Å. The estimated energy and $\left\langle {\mathrm{n̂}}_{1\alpha }{\mathrm{n̂}}_{10\beta }\right\rangle$ during the optimization process are shown in Figure 9 for two different sample sizes, *N*_*s*_ = 10^4^ and *N*_*s*_ = 10^5^. For small *N*_*s*_, Figure 9a,c reveals that the Quantum (*R*_*e*_ = 2.0 Å) and Quantum (hopping) proposals significantly outperform classical proposals. Similar to the case for FHMs, VMC with classical proposals all fail to converge to the correct ground state and $\left\langle {\mathrm{n̂}}_{1\alpha }{\mathrm{n̂}}_{10\beta }\right\rangle$ for *N*_*s*_ = 10^4^. Only when *N*_*s*_ is increased to 10^5^ do classical proposals begin to converge to the correct results. These results are consistent with the findings for FHMs, and underscore the potential of QA-VMC to accelerate VMC for molecular systems.

**Figure 9:**
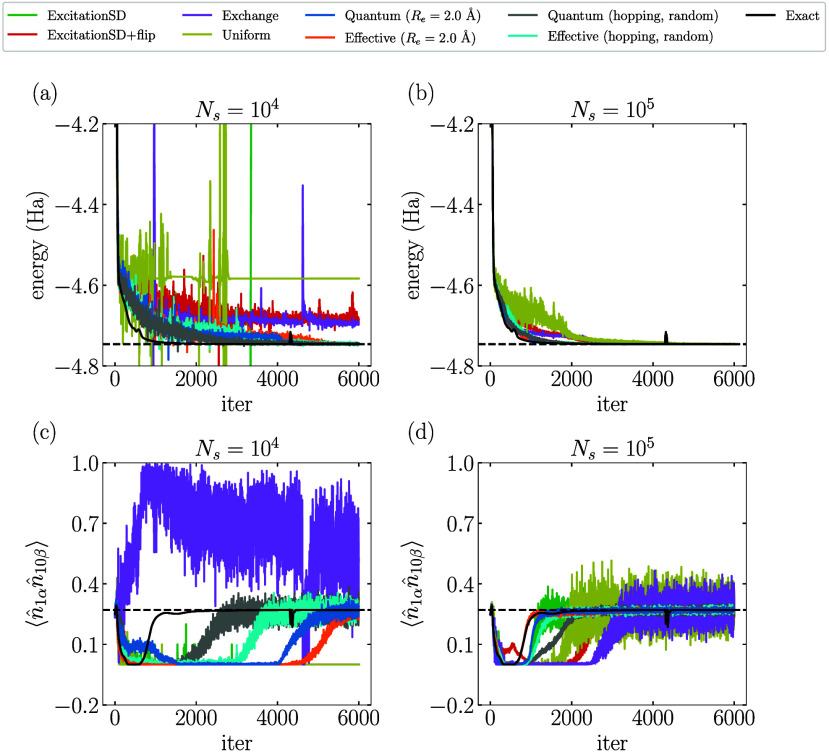
VMC optimization process of different proposals using the RBM (α = 3) ansatz for the hydrogen chain H_10_ with *R* = 2.0 Å: (a,b) energy; (c,d) $\left\langle {\mathrm{n̂}}_{1\alpha }{\mathrm{n̂}}_{10\beta }\right\rangle$ . Black solid lines in (a) and (b) represent the optimization trajectory using the exact gradients without sampling. Black dashed lines represent the exact ground-state energy in (a) and (b) or $\left\langle {\mathrm{n̂}}_{1\alpha }{\mathrm{n̂}}_{10\beta }\right\rangle$ for the exact ground state in (c) and (d).

#### Water Molecule

In addition to hydrogen chains, we also consider the water molecule as an example of more realistic systems. Here, we fix the bond angle at *∠*H–O–H = 104.5° and vary the O–H bond length *R*. As *R* increases, it also exhibits a transition from a weakly correlated system to a strongly correlated system. Figure 10a,b shows the absolute spectral gap δ obtained using various proposals for the ground state of H_2_O at different *R* using the OAOs and canonical molecular orbitals (CMOs). The results indicate that the Quantum and Effective proposals incorporating hopping terms outperform the classical proposals, whereas the classical proposals exhibit better performance than the Quantum and Effective proposals without hopping. This behavior can be attributed to the heterogeneous atomic composition of the system, because in such case some high-energy excited states are dominated by a very few configurations. Consequently, as shown by eq 17, if a configuration is transition to an excited state dominated by a very few configurations, then it is hard to transit to other configurations by the Quantum proposals, and the mixing time is therefore increased. The detailed mechanism is discussed in the Supporting Information. Including the hopping term in the effective Hamiltonian can increase the transition probability, thereby substantially enhancing the absolute spectral gap δ. Figure 10c,d further reveals that the absolute spectral gap δ obtained from the Quantum (hopping, γ = 0.9) proposal is significantly larger than that from the Quantum (hopping, γ = 0.1) proposal. This suggests that for H_2_O, increasing the proportion of hopping terms in the one-body part of the effective Hamiltonian is beneficial for enhancing the absolute spectral gap. In addition, the Quantum (hopping, random) proposal also achieves a comparably favorable performance.

**Figure 10:**
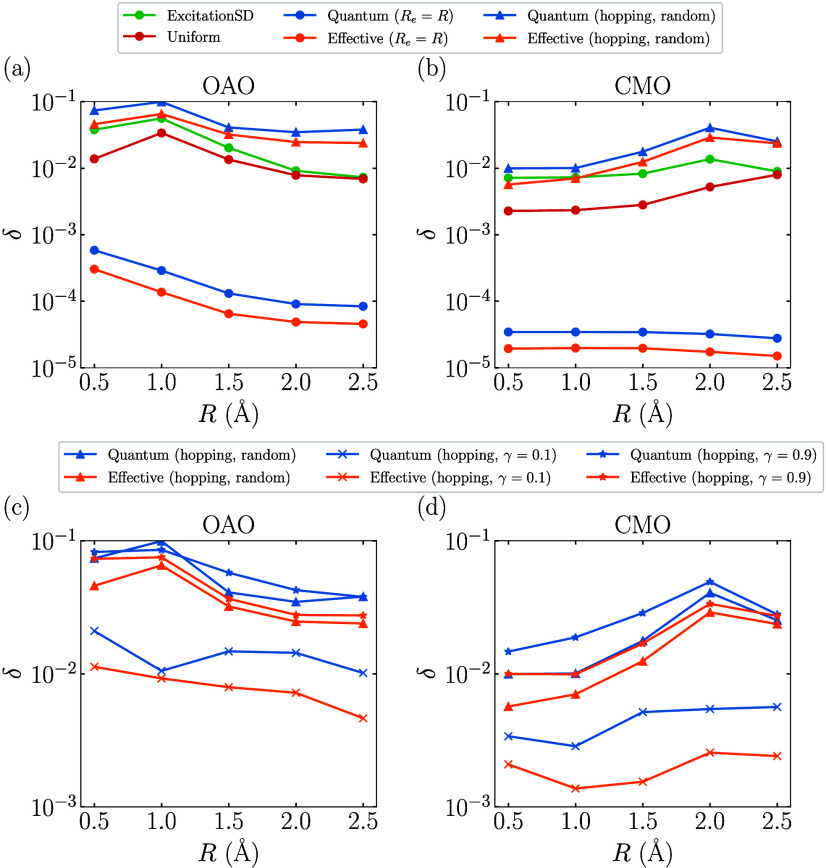
Absolute spectral gap δ obtained by diagonalizing the transition matrix P of each proposal for the ground state of H_2_O. For the Quantum proposal, δ is obtained as the maximal absolute spectral gap by scanning τ from 0.1 to 40.0 with a step size of 0.2. δ for the classical proposals, Quantum, and Effective proposals with or without the hopping term in the OAO (a) or CMO (b) basis. δ for the Quantum (hopping) and the Effective (hopping) proposals with different γ selections in the OAO (c) or CMO (d) basis.

Finally, we examine the performance of QA-VMC combined with the RBM ansatz (α = 3) in computing both the ground-state energy of H_2_O and an illustrative observable $\left\langle {\mathrm{n̂}}_{1\alpha }{\mathrm{n̂}}_{7\beta }\right\rangle$ at a bond length *R* = 2.0 Å and a fixed bond angle *∠*H–O–H = 104.5°. The estimated energy and the expectation value $\left\langle {\mathrm{n̂}}_{1\alpha }{\mathrm{n̂}}_{7\beta }\right\rangle$ during the optimization process are shown in Figure 11 for two different sample sizes (*N*_*s*_ = 10^3^ and *N*_*s*_ = 10^4^). Figure 11a,b shows that the variational energy curves obtained from QA-VMC and conventional VMC using classical proposals exhibit no significant differences. In contrast, Figure 11c,d reveals that the estimation of $\left\langle {\mathrm{n̂}}_{1\alpha }{\mathrm{n̂}}_{7\beta }\right\rangle$ using the Quantum and Effective proposals with hopping terms is significantly more accurate than that obtained with classical proposals, featuring smaller fluctuations. Interestingly, the Quantum (*R*_*e*_ = 2.0 Å) proposal results in strong oscillations, whereas the Effective (*R*_*e*_ = 2.0 Å) proposal demonstrates the opposite behavior, achieving notably stable and accurate results. This performance appears to contradict the small absolute spectral gap δ observed for the Effective (*R*_*e*_ = 2.0 Å) proposal in Figure 10. However, this apparent contradiction arises because the dominant electronic configuration in the highest excited state of the Hamiltonian, where the five highest-energy orbitals are all doubly occupied, has a low probability under the VMC sampling when the optimization starts from a reasonable initial configuration. In contrast, the mixing time shown in eq 19, which measures the worst-case scenario, is affected by such a configuration.

**Figure 11:**
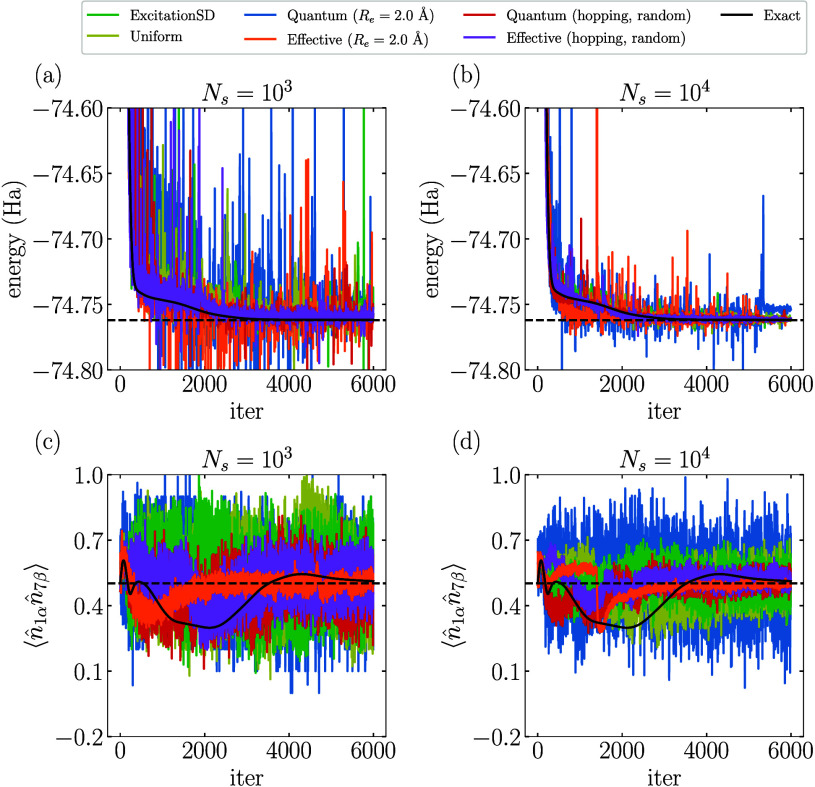
VMC optimization process of different proposals using the RBM (α = 3) ansatz for H_2_O at a bond length *R* = 2.0 Å: (a,b) energy; (c,d) $\left\langle {\mathrm{n̂}}_{1\alpha }{\mathrm{n̂}}_{7\beta }\right\rangle$ . Black solid lines in (a) and (b) represent the optimization trajectory without sampling. Black dashed lines represent the exact ground-state energy in (a) and (b) or $\left\langle {\mathrm{n̂}}_{1\alpha }{\mathrm{n̂}}_{7\beta }\right\rangle$ for the exact ground state in (c) and (d).

## Conclusion

In this work, inspired by the QeMCMC algorithm,^51^ originally designed for sampling classical Boltzmann distributions of spin models, we introduced the QA-VMC algorithm for solving the ground state of quantum many-body problems by leveraging the capabilities of quantum computers to enhance the sampling efficiency in VMC simulations. Pilot applications to FHMs and molecular systems reveal that the Quantum proposal exhibits larger absolute spectral gaps and reduced autocorrelation times compared to classical proposals, leading to more efficient sampling and faster convergence to the ground state in the VMC. This advantage is found to be especially pronounced for specific parameter ranges, where the ground-state configurations are concentrated in some dominant configurations separated by large Hamming distances. Besides, we also identified limitations of the introduced Quantum proposal, particularly when the system parameters approach some extreme values, leading to reducible Markov chains and vanishing absolute spectral gaps. To mitigate these issues, we proposed fixing certain parameters in the Hamiltonian used for time evolution in the Quantum proposal, which can maintain a nonzero absolute spectral gap and exhibit advantages over classical proposals across a wider range of system parameters and sizes. Furthermore, to extend the applicability of QA-VMC for molecular systems, we propose the Quantum (hopping) proposal, which incorporates additional hopping terms into the molecular Hamiltonian. This approach offers greater generality for molecular systems and is capable of delivering results comparable to those of the Quantum proposal for hydrogen chains. Our results suggest that QA-VMC has the potential to enhance the performance of VMC algorithms for large systems. By providing samples of good quality, fewer samples can be used in the VMC optimization, which also reduces the computational cost for the evaluation of local energy and gradients.

Future work will focus on further optimizing the Quantum proposal, including the automatic optimization of the evolution time, the use of Trotter decomposition or other Hamiltonian simulation techniques, and investigating the performance of QA-VMC on noisy quantum simulators and real quantum hardware. Additionally, exploring the application of QA-VMC to other quantum systems with more complex Hamiltonians will be crucial for assessing its broader applicability and potential for quantum advantage.

## Supplementary Material



## Notes

Time-averaged Quantum proposal versus Effective proposal, the absolute spectral gap of the Quantum proposal as a function of the evolution time, exact ground state distributions of the investigated models, and additional results for Fermi–Hubbard models, hydrogen chains, and H_2_O (PDF)
