# Supplementary material for: Quantum-Assisted Variational Monte Carlo
Source: Precis Chem. 2025 Jun 7;3(9):541–53. doi: 10.1021/prechem.5c00025 (PMC12458056; doi:10.1021/prechem.5c00025)
Supplement: Supplementary file 1 [file pc5c00025_si_001.pdf]

Supporting Information for "Quantum-assisted variational Monte Carlo"

Longfei Chang, Zhendong Li,* and Wei-Hai Fang
Key Laboratory of Theoretical and Computational Photochemistry, Ministry of Education, College of Chemistry, Beijing Normal University, Beijing 100875, China

I. TIME-AVERAGED QUANTUM PROPOSAL VERSUS EFFECTIVE PROPOSAL

To further investigate the relation between the Quantum and Effective proposals, we introduce the time-averaged Quantum proposal $\mathcal{Q}^{\mathrm{q(aver)}}(\boldsymbol{S}_i, \boldsymbol{S}_j; x_e, \tau)$

$$\mathcal{Q}^{\mathrm{q(aver)}}(\boldsymbol{S}_i, \boldsymbol{S}_j; x_e, \tau) = \frac{1}{2\tau}\int_{-\tau}^{+\tau} \mathcal{Q}^{\mathrm{q}}(\boldsymbol{S}_i, \boldsymbol{S}_j; x_e, \tau')\mathrm{d}\tau', \tag{1}$$

We calculate the absolute spectral gap δ for $\mathcal{Q}^{\mathrm{q(aver)}}(\boldsymbol{S}_i, \boldsymbol{S}_j; x_e, \tau)$ as a function of τ for 8-site FHMs and H_8, see Fig. S1. The results show that δ for $\mathcal{Q}^{\mathrm{q(aver)}}$ converge to that for the Effective proposals. The convergence for $R = 2.5$ Å in H_8 is much slower than that for smaller R.

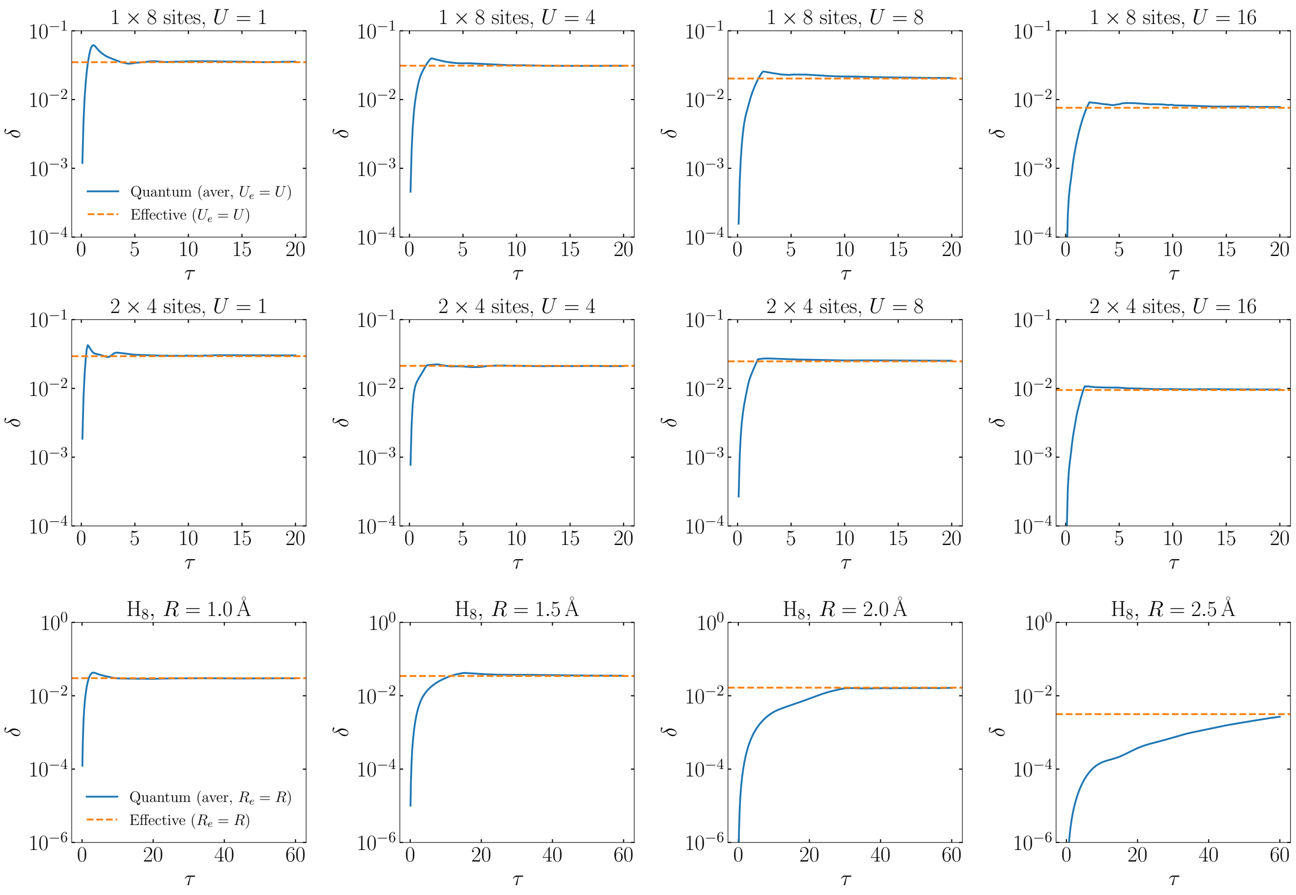

FIG. S1: Computed absolute spectral gap δ for the time-averaged Quantum proposal $\mathcal{Q}^{\mathrm{q(aver)}}(\boldsymbol{S}_i, \boldsymbol{S}_j; U_e, \tau)$ by diagonalizing the transition matrix $\mathcal{P}$ corresponding to the ground state of 8-site FHMs (Upper: 1×8, Middle: 2×4) with different U or the hydrogen chain H_8 (Lower) with different interatomic distance R as a function of τ. The dashed line represents the absolute spectral gap δ for the corresponding Effective proposal.

* zhendongli@bnu.edu.cn

II. A PRACTICAL IMPLEMENTATION OF THE QUANTUM (HOPPING) PROPOSAL WITH RANDOMIZED γ

The strategy to simulate the Quantum (hopping) proposal with randomized γ is similar to the Quantum-enhanced Markov chain Monte Carlo[1]. The Quantum (hopping, random) proposal used in our work can be described as

$$\mathcal{Q}^{\mathrm{q}}(\boldsymbol{S}_i, \boldsymbol{S}_j; \tau) = \frac{1}{\gamma_{\max} - \gamma_{\min}} \int_{\gamma_{\min}}^{\gamma_{\max}} \mathrm{d}\gamma \ \langle \boldsymbol{S}_j | \, e^{-\mathrm{i}\hat{H}(\gamma)\tau} \, |\boldsymbol{S}_i\rangle \, \langle \boldsymbol{S}_i | \, e^{\mathrm{i}\hat{H}(\gamma)\tau} \, |\boldsymbol{S}_j\rangle \tag{2}$$

where $\gamma_{\min} = 0.1$ and $\gamma_{\max} = 0.4$, respectively. For simplicity, γ is drawn from a discrete, rather than continuous, uniform distribution, such that the corresponding Quantum (hopping, random) proposal can be approximated to

$$\mathcal{Q}^{\mathrm{q}}(\boldsymbol{S}_i; \boldsymbol{S}_j, \tau) \approx \frac{1}{N+1} \sum_{k=0}^{N} \langle \boldsymbol{S}_j | \, e^{-\mathrm{i}\hat{H}(\gamma_{\min}+k\triangle\gamma)\tau} \, |\boldsymbol{S}_i\rangle \, \langle \boldsymbol{S}_i | \, e^{\mathrm{i}\hat{H}(\gamma_{\min}+k\triangle\gamma)\tau} \, |\boldsymbol{S}_j\rangle \tag{3}$$

where $\triangle\gamma = \frac{\gamma_{\max}-\gamma_{\min}}{N}$. In this work, we use $N = 6$. The corresponding Effective proposal is given by

$$\mathcal{Q}^{\mathrm{eff}}(\boldsymbol{S}_i, \boldsymbol{S}_j) = \frac{1}{N+1} \sum_{k=0}^{N} \sum_{n} p_n(\boldsymbol{S}_i; \gamma_{\min} + k\triangle\gamma) p_n(\boldsymbol{S}_j; \gamma_{\min} + k\triangle\gamma) \tag{4}$$

where $p_n(\boldsymbol{S}_i; \gamma_e) = |\langle \boldsymbol{S}_i | \Psi_n \rangle|^2$ and $\{|\Psi_n\rangle\}$ represents the eigenstates of $\hat{H}(\gamma_e)$.

III. THE ABSOLUTE SPECTRAL GAP δ OF THE QUANTUM PROPOSAL AS A FUNCTION OF THE EVOLUTION TIME τ

A. Hubbard model

We plot the absolute spectral gap δ of the Quantum proposal as a function of the evolution time τ in Fig. S2. It is seen that as the evolution time τ increases, δ reaches that of the Effective proposal δ_{eff} and then oscillates around it. The time required to reach δ_{eff} generally increases for large U in FHMs. Using a fixed parameter in the Quantum proposal ($U_e = 8$) reduces the evolution time required to surpass δ_{eff} in these cases.

B. Molecules

Fig. S3 shows the absolute spectral gap δ of the Quantum proposals, including the Quantum (hopping, random) proposal, as a function of the evolution time τ. As τ increases, δ approaches δ_{eff} and subsequently oscillates around it. The evolution time required to reach δ_{eff} increases gradually with the interatomic distance R in both hydrogen chains and the H_2O molecule. In the hydrogen chains, using a fixed parameter or adding the hopping term in the Quantum proposal ($R_e = 2.0$ Å or hopping) allows to reduce the evolution time for $R = 2.5$ Å.

IV. EXACT GROUND STATE DISTRIBUTIONS OF THE INVESTIGATED MODELS

Fig. S4 show the exact ground state distributions of 10-site FHMs (Top: 1×10, Second: 2×5) with different U and the hydrogen chain H_{10} in the OAO basis (upper) and H_2O in the OAO basis (middle) and CMO basis (lower) with different R to facilitate the discussion of the convergence of the MCMC algorithm with different proposals in the main text. It can be observed that as U in FHMs or R in H_n and H_2O increases, the ground-state distribution becomes more concentrated.

V. ADDITIONAL RESULTS FOR FHMS AND RANDOM FHMS

A. 1D FHMs

For comparison with the results shown in the main text, here we show the proposal probability distribution for an initial configuration $\mathcal{S}_i = (1, 1, -1, -1, \cdots, 1, 1, -1, -1)$ with a small ground-state probability (about 1.0×10^{-8}).

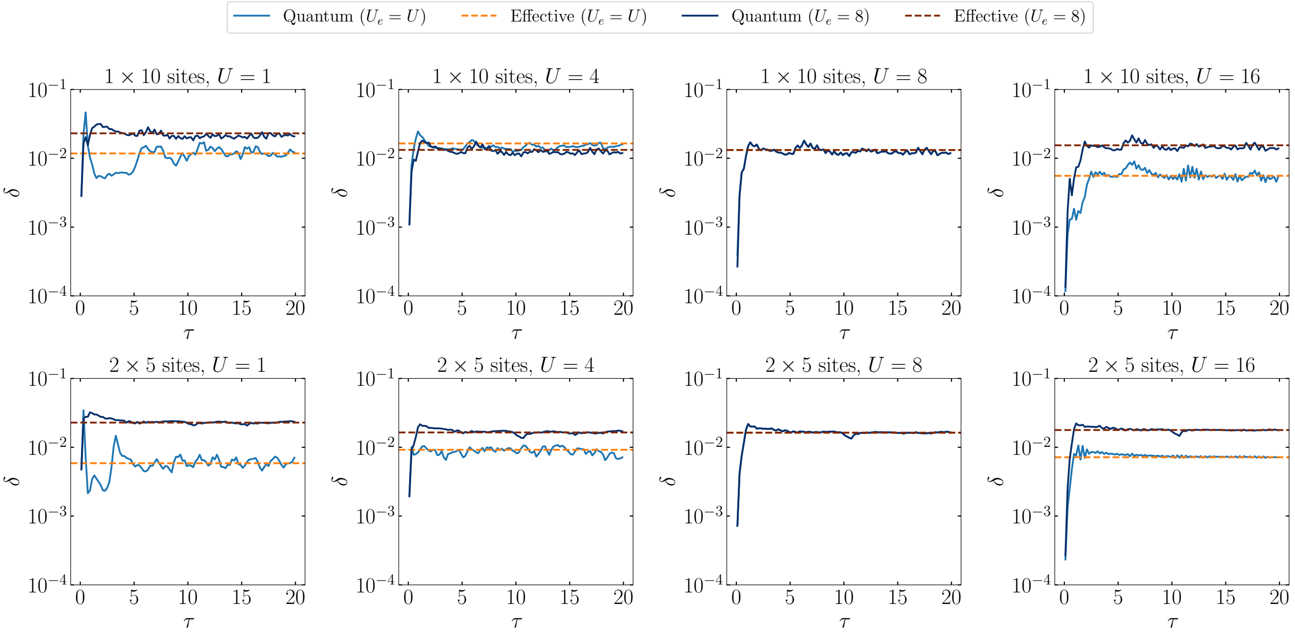

FIG. S2: The absolute spectral gap δ for the Quantum proposal obtained by diagonalizing the transition matrix $\mathcal{P}$ corresponding to the ground state of 10-site FHMs (Upper: 1×10, Lower: 2×5) with different U as a function of the evolution time τ. The dashed lines represent the absolute spectral gaps δ of the corresponding Effective proposal.

As shown in Fig. S5(a)-(c), the ExcitationSD, ExcitationSD+flip, and Exchange proposals generate configurations that move only by specific Hamming distance, consistent with the behavior observed in the main text. In contrast, the Quantum, Effective, and Uniform proposals enable transitions across unrestricted Hamming distances. However, both the Quantum and Effective proposals exhibit only a small probability of generating low-energy configurations, which contrasts with the behavior observed in the main text. This is because the initial configuration $\mathcal{S}_i$ has a very low ground-state probability. As a result, the product $p_0(\boldsymbol{S}_i)p_0(\boldsymbol{S}_j)$ contributes only negligibly to $\mathcal{Q}^{\text{eff}}(\boldsymbol{S}_i, \boldsymbol{S}_j)$.

B. 2D FHMs

Similar to the results for 1D FHMs in the main text, Fig. S6 shows the results obtained for 2D FHMs of ladder shapes. As shown in Fig. S6(a), most proposals approach a fixed value as U increases, except for the Exchange, Quantum ($U_e = U$), Effective ($U_e = U$) proposals. Interestingly, the absolute spectral gaps for the Quantum ($U_e = U$) and Effective ($U_e = U$) proposals show a non-monotonic decay, unlike the 1D case. Fig. S6(b) demonstrates that the Quantum proposals can also provide a large advantage over the classical proposals in 2D FHMs.

Figure S7 demonstrates that the evolution time τ required for δ of the Quantum proposal to first reach a fraction of δ_{eff} shows a very weak dependence of the system size in 2D FHMs of ladder shapes. This is in agreement with the findings in the main text for 1D FHMs.

C. Random FHMs

We further tested the behavior of the absolute spectral gap δ with different proposals averaged over FHMs with random parameters

$$\hat{H}(\{U\}) = -t \sum_{\langle i,j \rangle} \sum_{\sigma} (\hat{a}^{\dagger}_{i\sigma} \hat{a}_{j\sigma} + \text{h.c.}) + \sum_{i} U_i \hat{n}_{i\alpha} \hat{n}_{i\beta}, \tag{5}$$

where $t = 1$ and U_i is chosen from the uniform distribution $[8, 16]$. Fig. S8 shows the obtained results for 1D and 2D cases, respectively. The Exchange proposal has the smallest scaling among the classical proposals, but the value of δ is the smallest. The ratio of the scaling k between the ExcitationSD proposal and the Quantum ($U_e = U$) proposal is

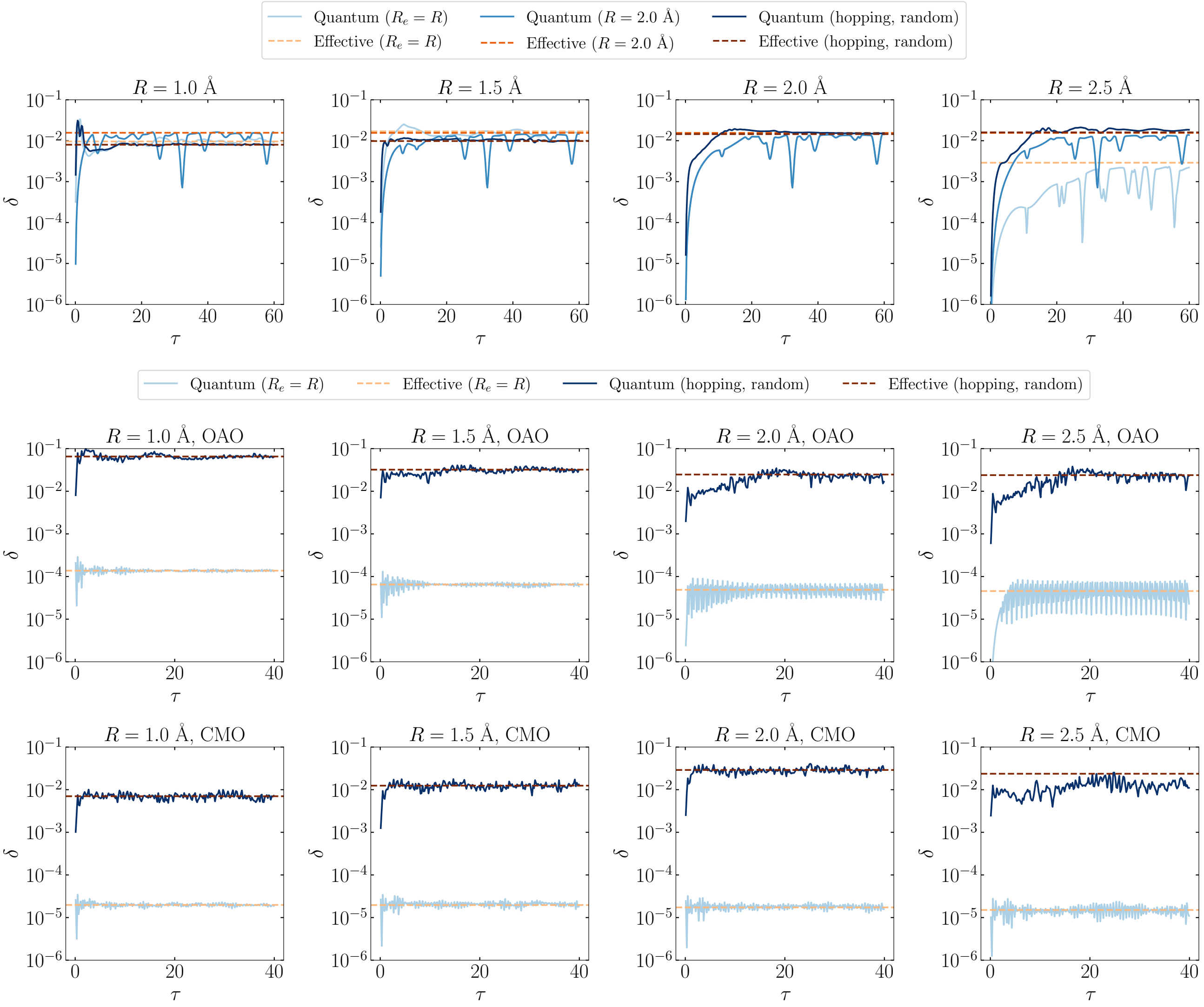

FIG. S3: The absolute spectral gap δ for the Quantum proposal obtained by diagonalizing the transition matrix $\mathcal{P}$ corresponding to the ground state of the hydrogen chain H_{10} (upper) and H_2O in the OAO basis (middle) and CMO basis (lower) as a function of the evolution time τ with different interatomic distance R. The dashed lines represent the absolute spectral gaps δ of the corresponding Effective proposal.

$0.96/0.24 \approx 4$ for 1D and $0.99/0.20 \approx 5$ for 2D, respectively, indicating a potentially large enhancement of the MCMC convergence by the Quantum proposals. Notably, using a fixed $U_e = 8$ performs well.

VI. ADDITIONAL RESULTS FOR THE HYDROGEN CHAINS

In Fig. S9, we further investigate the required evolution time τ for the Quantum ($R_e = R$, fixed $R_e = 2.0$ Å and hopping) proposals applied to hydrogen chains at various bond lengths. Detailed results for the absolute spectral gaps δ as a function of τ are provided in Fig. S3. As illustrated in Fig. S9, the time at which δ first exceeds $c\delta_{\text{eff}}$ (with $c = 0.6$, 0.7, and 0.8) increases slowly with system size, particularly for the Quantum proposal with a fixed R_e or the hopping term. Considering both the asymptotic behaviors of the absolute spectral gap and the required evolution time, we can conclude that the Quantum proposals have the potential to deliver an enhancement for the MCMC algorithm over classical proposals for large systems.

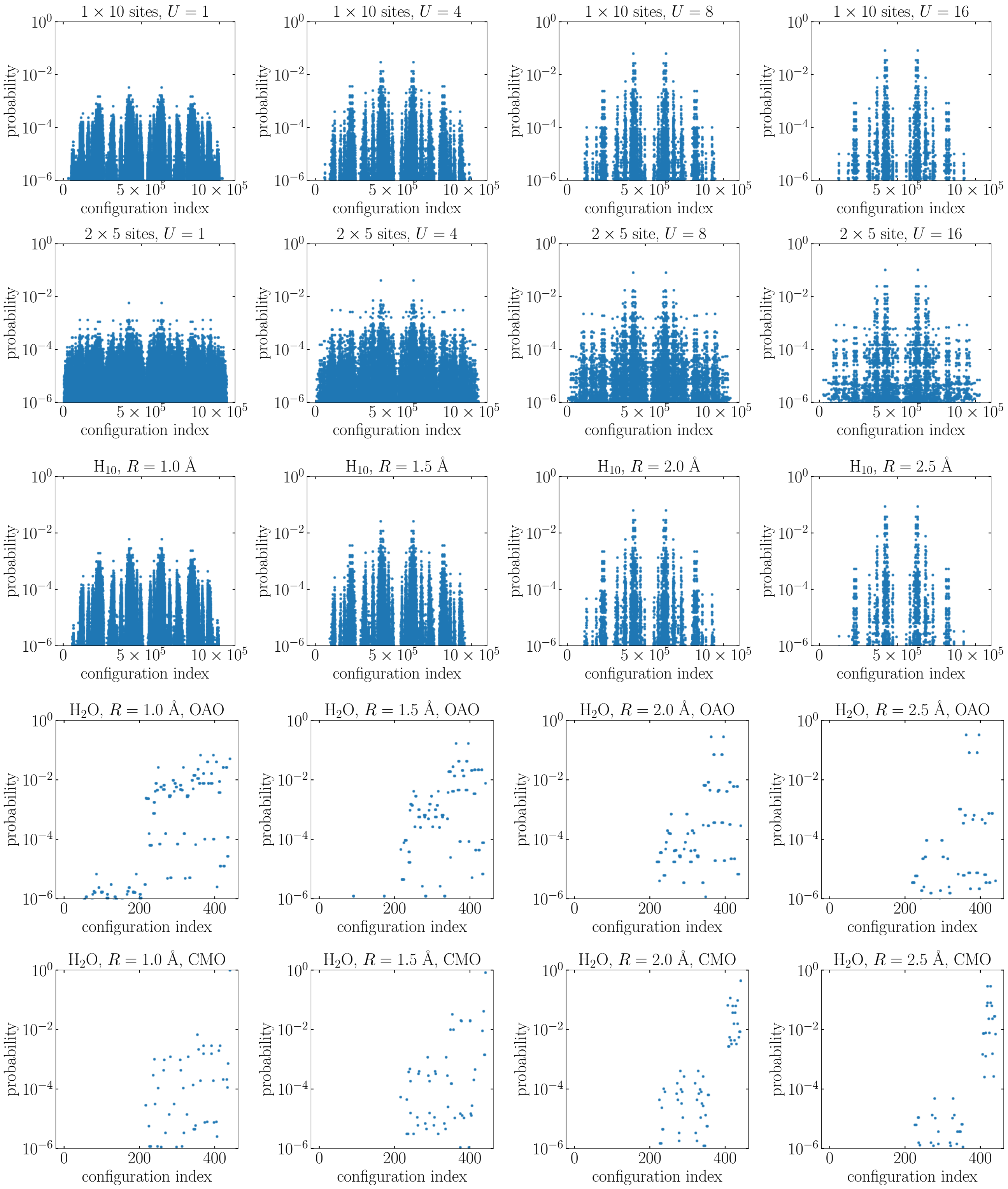

FIG. S4: The exact ground state distributions of 10-site FHMs (Top: 1×10; Second: 2×5) with different U, the hydrogen chain H_{10} in the OAO basis (Middle), and H_2O molecules (Bottom two rows) in the OAO (Fourth) and CMO (Bottom) basis with varying bond lengths R.

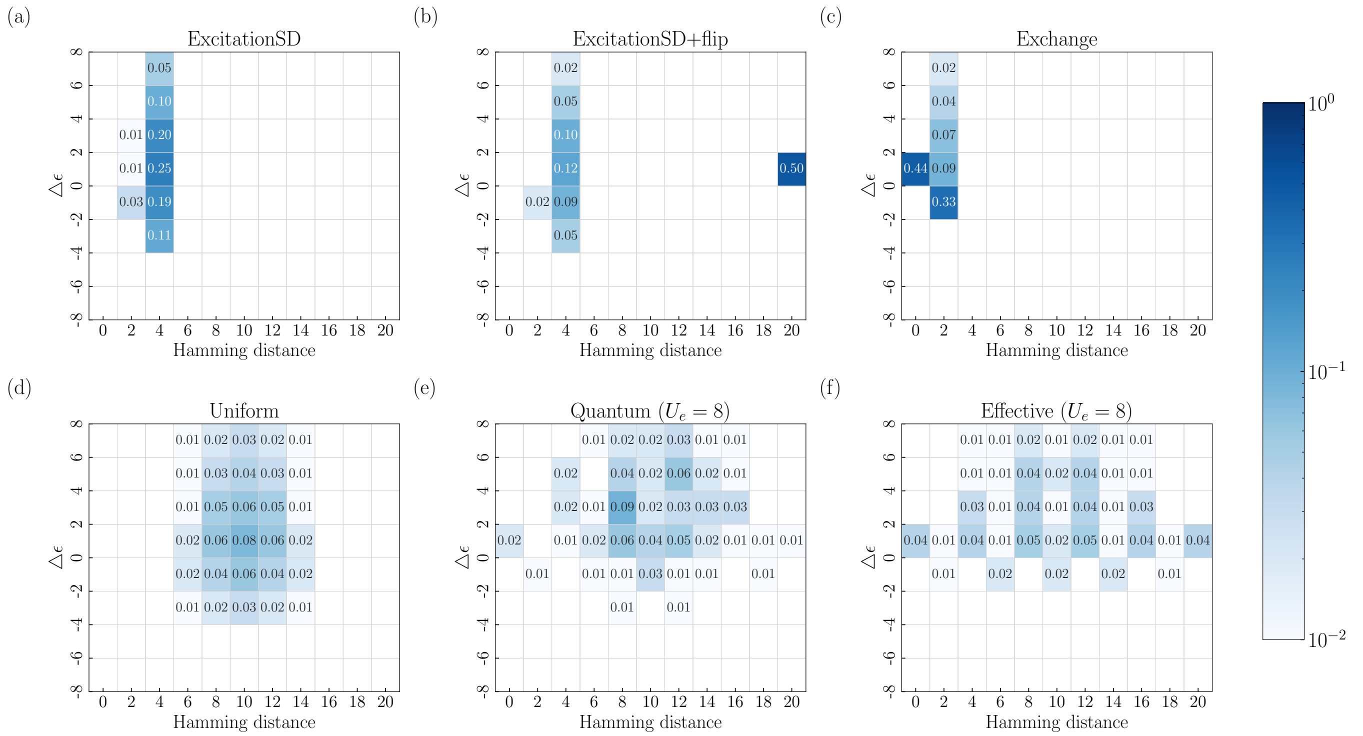

FIG. S5: Comparison of different proposal probabilities $\mathcal{Q}(\boldsymbol{S_i}, \cdot)$ from the qubit configuration $\boldsymbol{S}_i = (1, 1, -1, -1, \cdots, 1, 1, -1, -1)$ with a small ground-state probability in the 10-site 1D FHM with $U = 8$. (a)-(f) Two-dimensional histogram of $\mathcal{Q}(\boldsymbol{S_i}, \cdot)$ with the Hamming distance (between $\boldsymbol{S}_j$ and $\boldsymbol{S}_i$) and the 'energy' gap ($\triangle\epsilon = \log_{10}(P(\boldsymbol{S}_i)/P(\boldsymbol{S}_j))$ as the x and y axes, respectively. (a) ExcitationSD. (b) ExcitationSD+flip. (c) Exchange. (d) Uniform. (e) Quantum ($U_e = 8$). (f) Effective ($U_e = 8$).

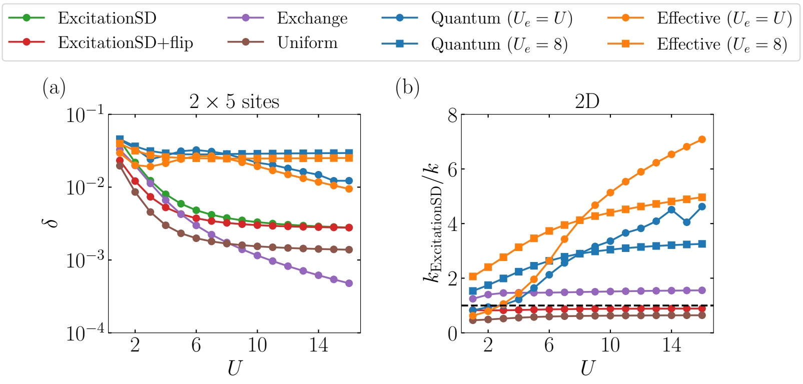

FIG. S6: The absolute spectral gap δ with different proposals for 2D FHMs. For the Quantum proposals, δ is obtained as the maximal value by scanning τ from 0.1 to 20 with a step size of 0.2. (a) δ with different proposals as a function of U for a 2D 10-site FHM (2×5). (b) $k_{\mathrm{ExcitationSD}}/k$ as a function of the parameter U, where k is the fitted exponent for δ as a function of the system size N. The black dashed line represents $k_{\mathrm{ExcitationSD}}/k = 1.0$.

VII. ADDITIONAL RESULTS FOR H_2O

Here, we analyze the reasons for the small absolute spectral gap δ for the Quantum and Effective proposals at $R_e = 2.0$ Å. For the H_2O molecule using the OAO basis, the orbital ordering of OAO is given by $[\mathrm{O_{1S}}, \mathrm{O_{2S}}, \mathrm{O_{2P_x}}, \mathrm{O_{2P_y}}, \mathrm{O_{2P_z}}, \mathrm{H_{1S}}, \mathrm{H_{1S}}]$, where $\mathrm{O_{1S}}$ corresponds to the lowest orbital energy. The qubit configuration $\boldsymbol{S}_i = (1, 1, 1, 1, 1, -1, 1, -1, 1, 1, -1, 1, -1, 1)$ is one of the two configurations with the largest ground-state probability. In Fig. S10 (a), (b), and (d), the classical proposals and the Effective ($R_e = 2.0$ Å) proposal exhibit similar behavior to that observed in the Hubbard model. In

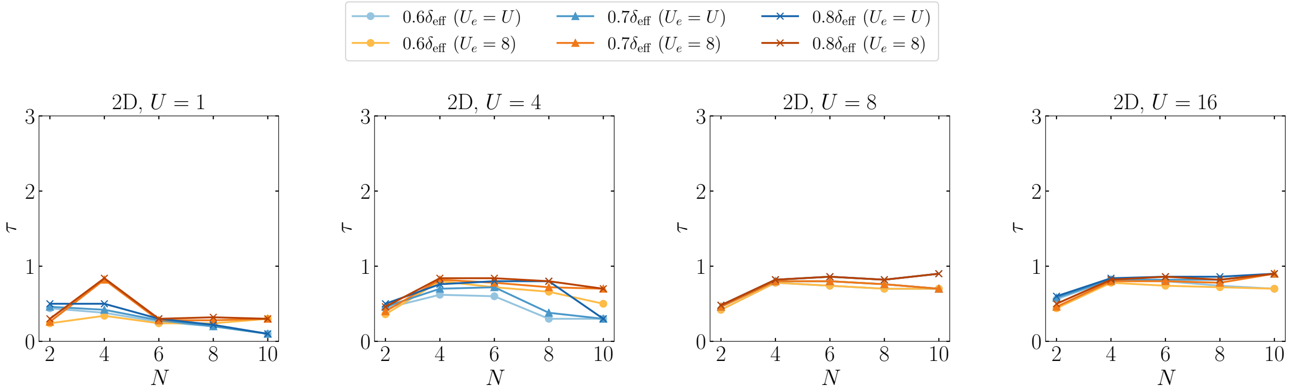

FIG. S7: The evolution time τ required for δ of the Quantum proposal to first reach $0.6\delta_{\rm eff}$, $0.7\delta_{\rm eff}$, and $0.8\delta_{\rm eff}$, respectively, as a function of N for the 2D FHMs ($2 \times N/2$ sites).

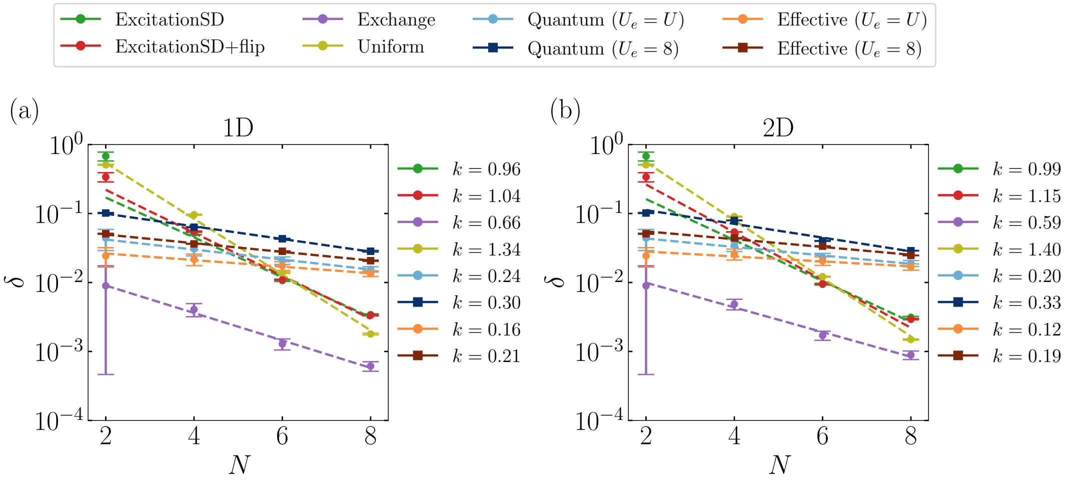

FIG. S8: The absolute spectral gap δ averaged over 100 random instances of N-site random FHMs as a function of the system size N: (a) 1D and (b) 2D of ladder shapes. The Quantum ($U_e = U$) proposal uses the corresponding random Hamiltonian for time evolution. For each random instance, δ is obtained as the maximal value by scanning τ from 0.1 to 20 with a step size of 0.1. Dashed lines represent the fitting results using the function $a2^{-kN}$.

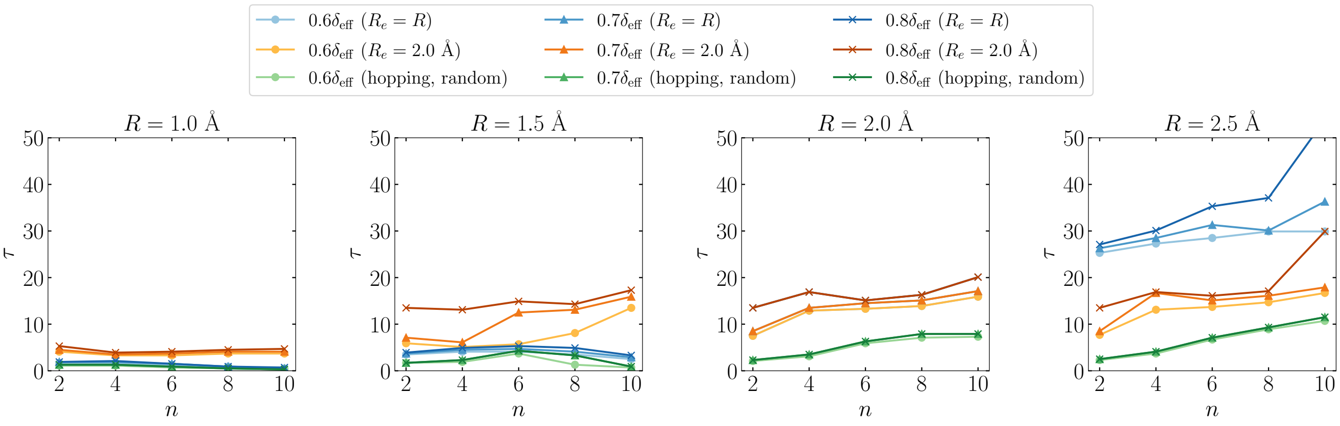

FIG. S9: The evolution time τ required for δ of the Quantum proposal to first exceeds $c\delta^{\rm eff}$ ($c = 0.6$, 0.7, and 0.8) as a function of the system size n for the ground state of hydrogen chains H_n with different interatomic distance R.

contrast, the Quantum ($R_e = 2.0$ Å) proposal tends to repeatedly generate the same configuration $\boldsymbol{S}_j = \boldsymbol{S}_i$, resulting in a 'lazy' Markov chain with limited state-space exploration.

For comparision, Fig. S11(d) shows that when $\boldsymbol{S}_i = (-1,-1,-1,-1,1,1,1,1,1,1,1,1,1,1)$, which has a small ground-state probability (approximately 1.2×10^{-10}), the Effective ($R_e = 2.0$ Å) proposal also exhibits a high probability of proposing no change, and thus lead to a small absolute spectral gap δ. Nevertheless, because this configuration has an extremely low ground-state probability, the VMC combined with the Effective ($R_e = 2.0$ Å) proposal can still achieve satisfactory results as shown in the main text. Upon incorporating the hopping term, as shown in Fig. S10 (e-f) and Fig. S11 (e-f), the Quantum proposal enables transitions across unrestricted Hamming distances with small 'energy' changes, regardless of the initial configuration. This mechanism contributes to a larger spectral gap δ compared with the Quantum proposal without hopping terms.

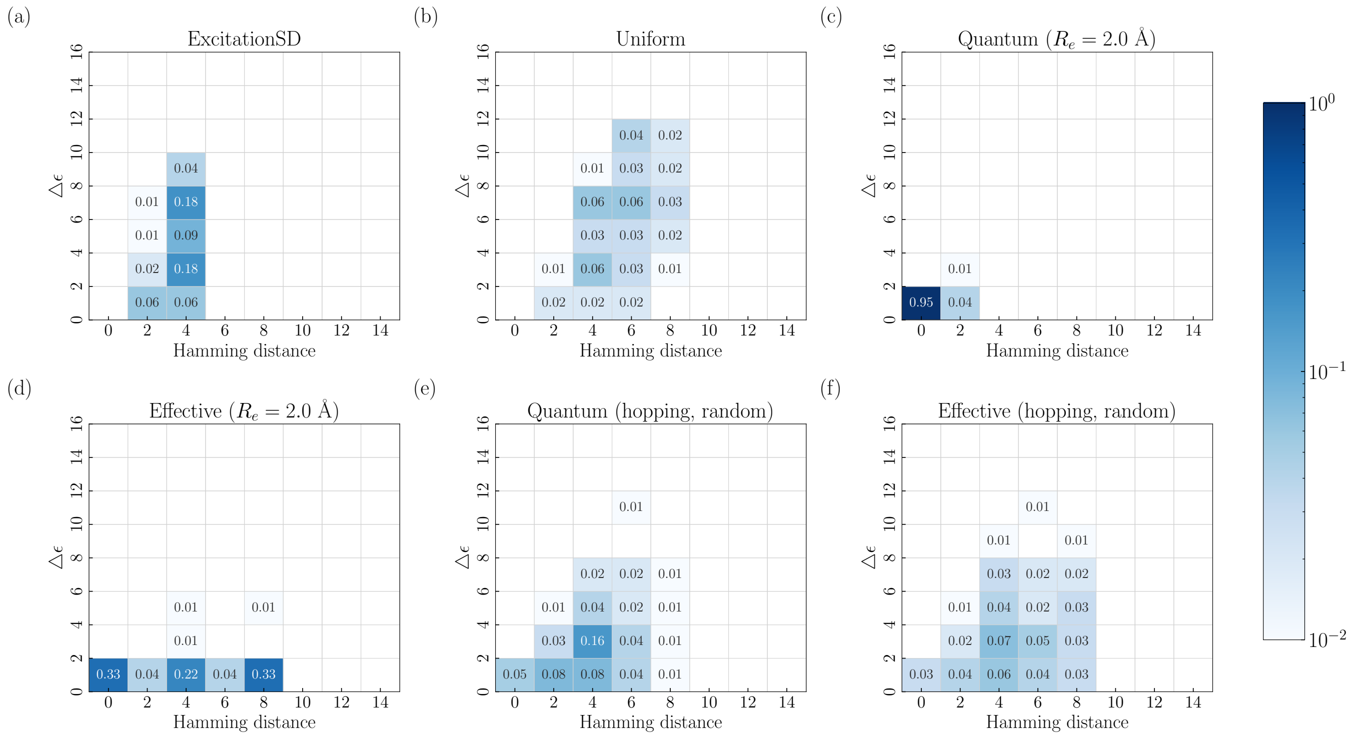

FIG. S10: Comparison of different proposal probabilities $\mathcal{Q}(\boldsymbol{S_i}, \cdot)$ from the qubit configuration $\boldsymbol{S}_i = (1,1,1,1,1,-1,1,-1,1,1,-1,1,-1,1)$ with the largest ground-state probability of H_2O in the OAO basis with $R = 2.0$ Å. (a)-(f) Two-dimensional histogram of $\mathcal{Q}(\boldsymbol{S_i}, \cdot)$ with the Hamming distance (between $\boldsymbol{S}_j$ and $\boldsymbol{S}_i$) and the 'energy' gap ($\triangle\epsilon = \log_{10}(P(\boldsymbol{S}_i)/P(\boldsymbol{S}_j))$ as the x and y axes, respectively. (a) ExcitationSD. (b) Uniform. (c) Quantum ($R_e = 2.0$ Å). (d) Effective ($R_e = 2.0$ Å). (e) Quantum (hopping, random). (f) Effective (hopping, random).

[1] D. Layden, G. Mazzola, R. V. Mishmash, M. Motta, P. Wocjan, J.-S. Kim, and S. Sheldon, Quantum-enhanced Markov chain Monte Carlo, Nature **619**, 282 (2023).

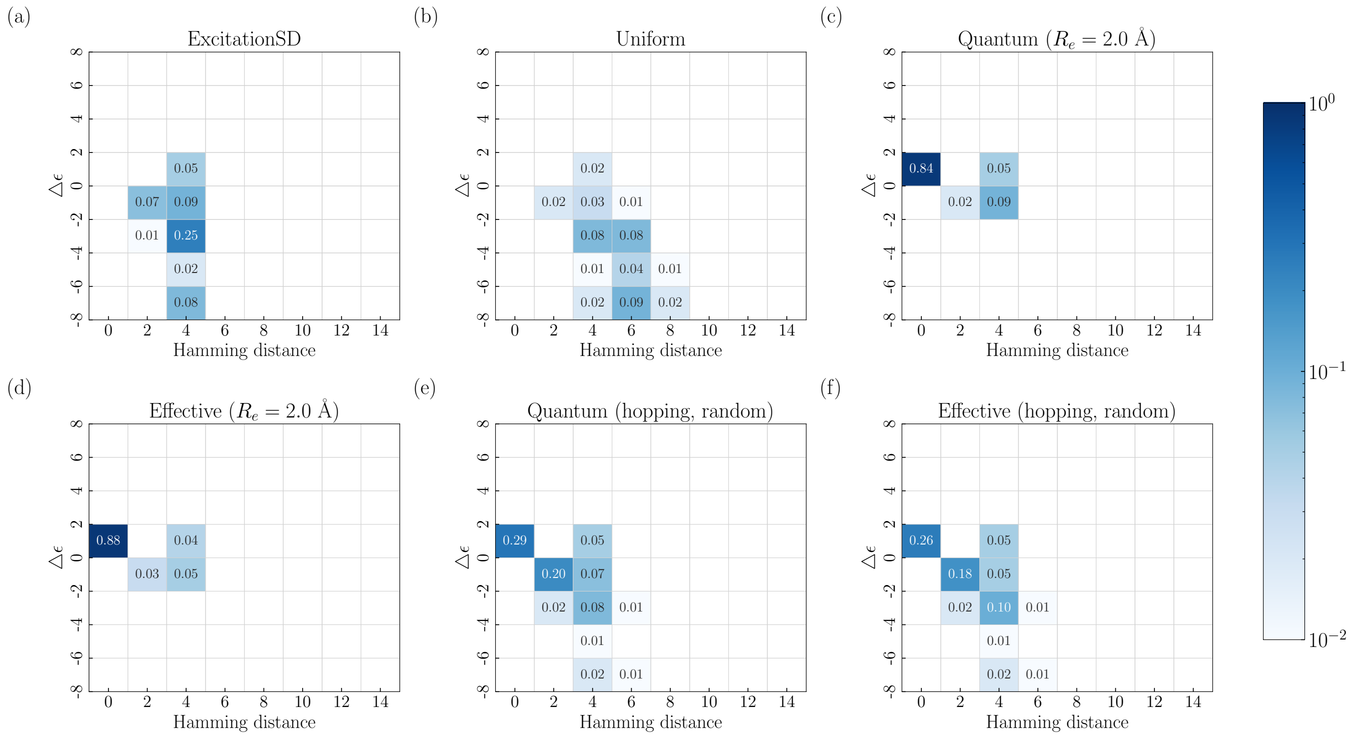

FIG. S11: Comparison of different proposal probabilities $\mathcal{Q}(\boldsymbol{S_i}, \cdot)$ from the qubit configuration $\boldsymbol{S}_i = (-1, -1, -1, -1, 1, 1, 1, 1, 1, 1, 1, 1, 1, 1)$ with a small ground-state probability of H_2O in the OAO basis with $R = 2.0$ Å. (a)-(f) Two-dimensional histogram of $\mathcal{Q}(\boldsymbol{S_i}, \cdot)$ with the Hamming distance (between $\boldsymbol{S}_j$ and $\boldsymbol{S}_i$) and the 'energy' gap ($\triangle\epsilon = \log_{10}(P(\boldsymbol{S}_i)/P(\boldsymbol{S}_j))$ as the x and y axes, respectively. (a) ExcitationSD. (b) Uniform. (c) Quantum ($R_e = 2.0$ Å). (d) Effective ($R_e = 2.0$ Å). (e) Quantum (hopping, random). (f) Effective (hopping, random).
